# Transcriptional regulation of *Caenorhabditis elegans* FOXO/DAF-16 modulates lifespan

**DOI:** 10.1186/2046-2395-3-5

**Published:** 2014-04-23

**Authors:** Ankita Bansal, Eun-Soo Kwon, Darryl Conte, Haibo Liu, Michael J Gilchrist, Lesley T MacNeil, Heidi A Tissenbaum

**Affiliations:** 1 Program in Gene Function and Expression, University of Massachusetts Medical School, Worcester, MA 01605, USA; 2 Laboratory of Cell Signaling, Aging Research Center, Korea Research Institute of Bioscience and Biotechnology, 125 Gwahak-ro, Yuseong-gu, Daejeon 306-809, Korea; 3 RNA Therapeutics Institute, University of Massachusetts Medical School, Worcester, MA 01605, USA; 4 MRC National Institute for Medical Research, The Ridgeway, Mill Hill, London, UK; 5 Program in Systems Biology, University of Massachusetts Medical School, Worcester, MA 01605, USA; 6 Program in Molecular Medicine, University of Massachusetts Medical School, Worcester, MA 01605, USA

**Keywords:** Longevity, DAF-16/FOXO, *C. elegans*, Transcription, Aging, Isoforms

## Abstract

**Background:**

Insulin/IGF-1 signaling plays a central role in longevity across phylogeny. In *C. elegans*, the forkhead box O (FOXO) transcription factor, DAF-16, is the primary target of insulin/IGF-1 signaling, and multiple isoforms of DAF-16 (a, b, and d/f) modulate lifespan, metabolism, dauer formation, and stress resistance. Thus far, across phylogeny modulation of mammalian FOXOs and DAF-16 have focused on post-translational regulation with little focus on transcriptional regulation*.* In *C. elegans*, we have previously shown that DAF-16d/f cooperates with DAF-16a to promote longevity. In this study, we generated transgenic strains expressing near-endogenous levels of either *daf-16a* or *daf-16d/f*, and examined temporal expression of the isoforms to further define how these isoforms contribute to lifespan regulation.

**Results:**

Here, we show that DAF-16a is sensitive both to changes in gene dosage and to alterations in the level of insulin/IGF-1 signaling. Interestingly, we find that as worms age, the intestinal expression of *daf-16d/f* but not *daf-16a* is dramatically upregulated at the level of transcription. Preventing this transcriptional upregulation shortens lifespan, indicating that transcriptional regulation of *daf-16d/f* promotes longevity. In an RNAi screen of transcriptional regulators, we identify *elt-2 (*GATA transcription factor) and *swsn-1* (core subunit of SWI/SNF complex) as key modulators of *daf-16d/f* gene expression. ELT-2 and another GATA factor, ELT-4, promote longevity via both DAF-16a and DAF-16d/f while the components of SWI/SNF complex promote longevity specifically via DAF-16d/f.

**Conclusions:**

Our findings indicate that transcriptional control of *C. elegans* FOXO/*daf-16* is an essential regulatory event. Considering the conservation of FOXO across species, our findings identify a new layer of FOXO regulation as a potential determinant of mammalian longevity and age-related diseases such as cancer and diabetes.

## Background

The evolutionarily conserved insulin/IGF-1 signaling (IIS) pathway regulates lifespan from worms to mammals and in *C. elegans* also modulates dauer formation, stress response, and metabolism [1-4]*.* The *C. elegans* IIS pathway consists of an insulin/IGF-1 receptor (DAF-2) [5], a PI 3-kinase (AGE-1/AAP-1) [6,7] serine/threonine kinases (PDK-1, AKT-1, and AKT-2) [8,9], and a Forkhead Box O (FOXO) transcription factor (DAF-16) [10,11]. IIS ultimately results in the AKT-dependent phosphorylation of DAF-16, thereby preventing DAF-16 from entering the nucleus to regulate its target genes [3,4,12]. Therefore, the *C. elegans* FOXO ortholog, DAF-16 is the primary downstream target of the IIS pathway [10,11].

In contrast to *C. elegans*, mammals have three closely related FOXO proteins (FOXO1, FOXO3, and FOXO4) that share a high degree of homology, overlapping expression patterns, and common target genes [13,14]. These FOXOs appear to have discrete functions as each mutant displays a distinct phenotype: *Foxo1* null mice die as embryos due to defects in angiogenesis [15], *Foxo3* mutants are viable but show age-dependent female sterility [15,16], and *Foxo4* null mutants are viable with no detectable phenotype [15]. Interestingly, conditional somatic deletion of all three *Foxo* loci results in a cancer-prone phenotype that includes age-progressive thymic cancers and hemangiomas, indicating that *Foxo1*, *Foxo3*, and *Foxo4* are redundant tumor suppressor genes specifically involved in endothelial growth suppression [17]. Recently, a more distantly related FOXO family member, FOXO6, was shown to regulate gluconeogenesis [18]. Thus, four mammalian FOXO homologs play distinct and overlapping roles in multiple biological processes.

**Figure 1:**
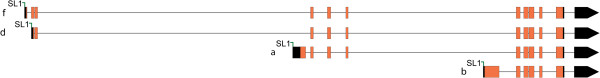
**Structure of *daf-16 *isoforms.** Coding regions are orange-filled boxes, and introns are lines. The SL1 trans-spliced region is indicated with a green line. The SL1 trans-spliced reads for the different isoforms are: *daf-16d/f*, 8 reads; *daf-16d*, 9 reads; *daf-16a*, 208 reads; *daf-16b*, 14 reads. See Additional file 1: Table S1 for ESTs associated with the different isoforms.

DAF-16, the only *C. elegans* FOXO ortholog*,* expresses multiple isoforms (Figure 1). Initial studies on DAF-16 identified three different transcripts, *daf-16a1*, *daf-16a2*, and *daf-16b. daf-16a1* and *daf-16a2* share the same promoter and 11 exons, but alternative splicing at exon 3 results in an insertion of two additional amino acids in the DAF-16a1 protein compared to DAF-16a2 [19,20]. *daf-16b* encodes a shorter isoform, expressed from a different promoter and results in a protein that includes the same C-terminal 319 amino acids as the DAF-16a1/a2 isoforms. Recently, we identified a third DAF-16 isoform, *daf-16d/f*, which is driven by a distinct promoter and initiates approximately 10 kb upstream of *daf-16a1/a2. daf-16d/f* is encoded by 14 exons, 10 of which are shared with DAF-16a1/a2 and DAF-16b. Studies using isoform-specific RNAi and transgenes have revealed cooperative as well as specific functions for these three DAF-16 isoforms (DAF-16a, DAF-16b, and DAF-16d/f) in the regulation of lifespan, dauer formation, fat storage, and stress resistance [21]. For lifespan regulation, however, we found that only DAF-16a and DAF-16d/f are involved [21]. The relative contribution of each of these two isoforms and regulation of each isoform remains unknown.

To date, much has been learned about the post-translational regulation of FOXO/DAF-16. For example, FOXO/DAF-16 is directly phosphorylated by kinases including AKT-1, AKT-2, SGK-1, Jun-N-terminal kinase (JNK/JNK-1) [22-24], Ste20-like protein kinase (MST1/CST-1) [25], and AMP-activated protein kinase (AMPK/AAK-1) [26,27], and FOXO/DAF-16 interacts with a Serine/threonine-protein phosphatase 4 regulatory subunit, SMK-1 [28]. FOXO/DAF-16 also interacts with 14-3-3 proteins, which modulate the ability of FOXO/DAF-16 to interact with cofactors, including the sirtuin family (SIRT1/SIR-2.1) of NAD-dependent deacetylases [3,4,29,30]. These interactions and direct post-translational modifications function to modulate the activity of FOXO/DAF-16 under different conditions [3,4].

Despite the growing body of knowledge centered on the post-translational regulation of FOXO/DAF-16, little is known about the transcriptional regulation of *daf-16* in *C. elegans*. To begin to address these issues, we examined the expression of the *daf-16a* and *daf-16d/f* isoforms throughout development and adulthood. We found that *daf-16d/f* expression is dramatically increased at the level of transcription during the young adult stage, and this upregulation of *daf-16d/f* expression is required for longevity. Furthermore, using an RNAi screen we identified two transcriptional regulators of *daf-16d/f* expression: *elt-2*, encoding an essential GATA transcription factor required for intestinal development [31,32], and *swsn-1*, encoding a core component of the SWI/SNF chromatin remodeling complex [33,34]. ELT-2 and another GATA factor, ELT-4, are required for the expression of both *daf-16a* and *daf-16d/f* and for the ability of both *daf-16a* and *daf-16d/f* to promote longevity. By contrast, SWSN-1 and other SWI/SNF components are required for the upregulation of *daf-16d/f* expression in the intestine of young adults, but not *daf-16a* expression. Consistent with this finding, components of the SWI/SNF complex promote longevity via *daf-16d/f* but not *daf-16a*. Taken together, our findings reveal that transcriptional regulation of *daf-16d/f* is an important regulatory event in lifespan determination.

## Results and discussion

### *daf-16* has multiple isoforms

*daf-16* functions as a central regulator of multiple biological processes including lifespan, development, fat storage, and stress resistance [1-4]. The *C. elegans* genome resource, Wormbase (http://www.wormbase.org), predicts eight putative isoforms of *daf-16*, with each isoform designated by a lowercase letter following the cosmid name for *daf-16* (R13H8.1a-h). To verify expression of the *daf-16* isoforms, we first analyzed the Gurdon Institute *C. elegans* expressed sequence tag (EST) database (http://genomics.nimr.mrc.ac.uk/online/worm-fl-db.html) [35,36]. As shown in Additional file 1: Table S1, ESTs were identified that map to *daf-16f* (R13H8.1f), *daf-16d* (R13H8.1d), *daf-16a* (R13H8.1b/c), and *daf-16b* (R13H8.1a). In addition to ESTs, we examined published capped RNA (Cap-Seq) (Gu *et al.*[37]), total mRNA, and ribosome-associated mRNA deep sequencing datasets (Stadler *et al.*[38]). These datasets support the existence of *daf-16f*, *daf-16d*, *daf-16a1*, *daf-16a2*, and *daf-16b*. In particular, the Cap-Seq data specifically identified four spliced leader (SL1) sites corresponding to the capped 5’ ends of *daf-16f*, *daf-16d*, *daf-16a1/a2*, and *daf-16b*, as indicated in green in Figure 1. Therefore, our findings verify the existence of five isoforms in contrast to eight that were predicted initially for DAF-16 - (DAF-f (R13H8.1f), DAF-16d (R13H8.1d), DAF-16a1 (R13H8.1b), DAF-16a2 (R13H8.1c), and DAF-16b (R13H81.a).

**Figure 2:**
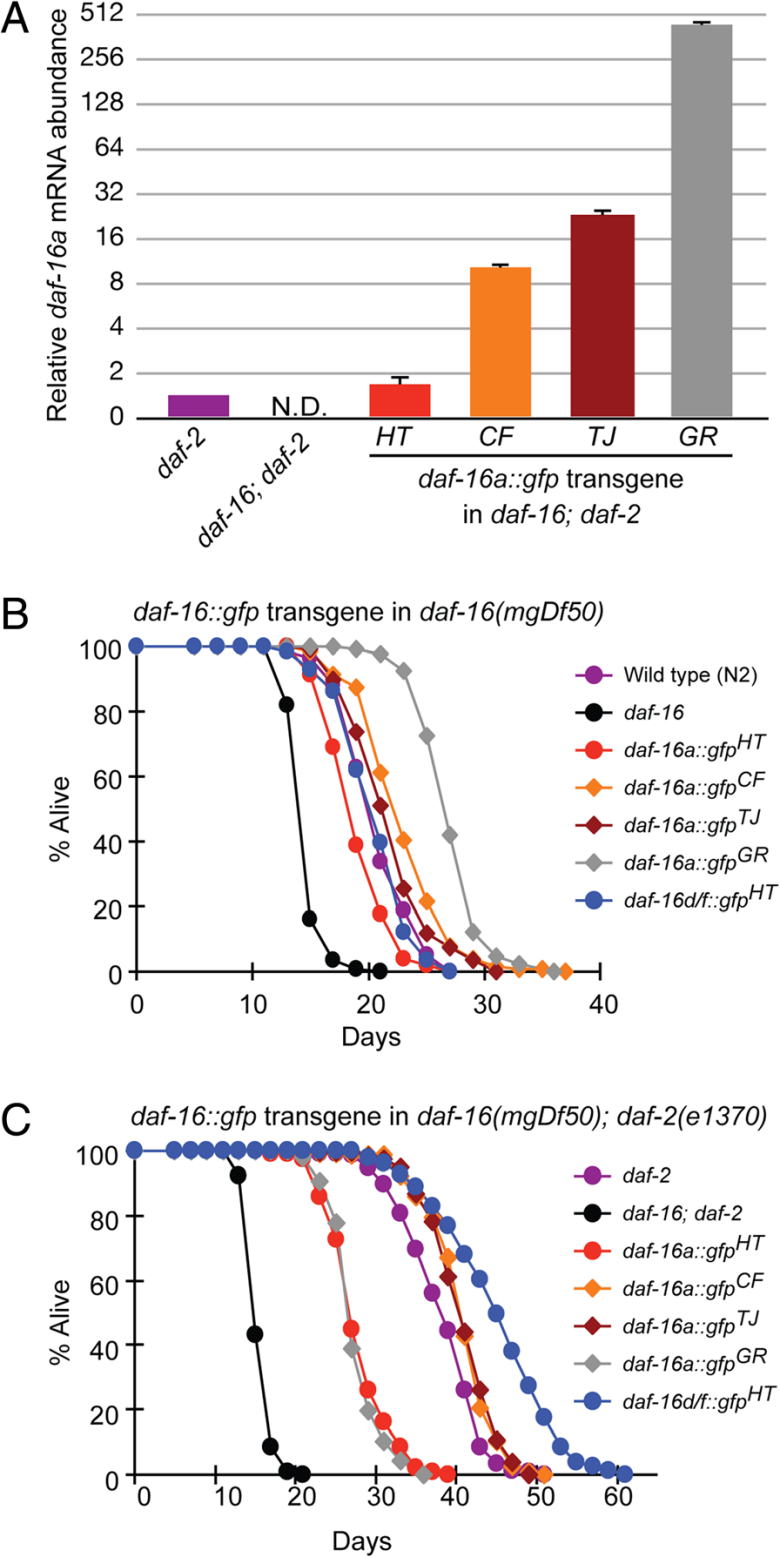
**Effects of altering DAF-16a dosage on lifespan. (A)** Expression levels of *daf-16a* mRNA in various *daf-16(mgDf50)*; *daf-2(e1370)*; *daf-16a::gfp* strains were compared to that of *daf-2(e1370)* worms. The graph is plotted on a log2 scale. Error bars represent standard deviation (S.D.) from two independent repeats. Statistical values are given in Additional file 1: Table S2. **(B)** Lifespan of *daf-16(mgDf50)* worms carrying various *daf-16a::gfp* transgenes as well as low-copy *daf-16d/f::gfp*^*HT*^. Three high-copy *daf-16a* transgenic worms live longer than wild type. *daf-16a::gfp*^*HT*^ worms lived shorter than wild-type, while the lifespan of the *daf-16d/f::gfp*^*HT*^ strain is comparable to wild type. **(C)** Lifespan of *daf-16(mgDf50)*; *daf-2(e1370)* worms carrying various *daf-16::gfp* transgenes. *daf-16a::gfp*^*HT*^ worms lived shorter than the *daf-2(e1370)* mutants. *daf-16(mgDf50)*; *daf-2(e1370)*; *daf-16a::gfp*^*GR*^ worms had the shortest lifespan among the high-copy *daf-16a* transgenic strains. *daf-16d/f::gfp*^*HT*^ transgene alone fully rescued the lifespan extension of *daf-2(e1370)* mutants. One lifespan experiment is shown from a total of three repeats; each with similar results. All lifespan data are shown in Additional file 1: Table S3.

Previous RNAi and transgenic studies examined the function of different *daf-16* isoforms (*daf-16a*, *b*, *d/f*) [19-21], which revealed that *daf-16b* functions in dauer formation, while *daf-16a* and *daf-16d/f* contribute to lifespan regulation. According to our previous studies [21], *daf-16a* constructs could not rescue the lifespan phenotype of a complete loss of *daf-16*, and *daf-16d* transgenes were unstable and did not consistently rescue *daf-16* loss-of-function. However, transgenes expressing the *daf-16f* isoform or *daf-16d/f* showed similar expression patterns and consistently rescued the complete loss of *daf-16*[21] (Figure 2). Moreover, based on the CapSeq data, both *daf-16f* and *daf-16d* have the same transcription start site. Therefore, we continue to refer to these isoforms as *daf-16d/f* in this study.

### Effects of altering DAF-16a dosage on lifespan

Dissecting the relative contributions of DAF-16d/f and DAF-16a in the regulation of longevity has been challenging due to incomplete knockdown by isoform-specific RNAi [21] and the lack of isoform-specific mutants. This is further complicated by the existence of a dose-dependent effect of *daf-16a* and *daf-16d/f* transgenes [21]. To expand our analysis of the *daf-16* dosage effect on lifespan, we first generated a new low-copy *daf-16a* transgene by microparticle bombardment, which we refer to as *daf-16a:gfp*^*HT*^. In addition, we chose three commonly used high-copy *daf-16a* transgenes, referred to as *daf-16a::gfp*^*CF*^ derived from strain CF1407 [20], *daf-16a::gfp*^*TJ*^ from TJ356 [39], and *daf-16a::gfp*^*GR*^ from RX86 [40]. For all the analyses that follow, we first placed each *daf-16* transgene into the same genetic background which is a null allele of the endogenous *daf-16* locus [*daf-16(mgDf50)*].

To compare the levels of *daf-16a* in the different transgenic strains, we measured the level of *daf-16a* expression from each transgene in the *daf-16(mgDf50)*; *daf-2(e1370)* background and compared it to the endogenous level of *daf-16a* in *daf-2(e1370)* mutants (Figure 2A, Additional file 1: Table S2). The level of *daf-16a* expressed from the newly generated *daf-16a*^*HT*^ transgenic strain was approximately two-fold higher than the endogenous *daf-16a* in the *daf-2(e1370)* mutant, while the commonly used *daf-16a* transgenes were expressed at 10- to 400-fold higher levels than endogenous *daf-16a*. These results show the following order for levels of the *daf-16a* transcript: endogenous *daf-16a* < *HT* < *CF* < *TJ* < <*GR*. Consistent with the mRNA expression data, the level of DAF-16a protein from the transgenic strains also increases (Additional file 1: Figure S1). The GFP intensity in the four transgenic strains correlated with the level of expression of DAF-16a (data not shown).

Next, we determined the nuclear:cytosolic ratio of DAF-16a in the transgenic strains expressing different levels of DAF-16a (Additional file 1: Figure S2) in *daf-2(e1370)* background. DAF-16a was almost entirely in the nucleus when expressed at low levels from the *daf-16a::gfp*^*HT*^ transgene. By contrast, DAF-16a was mainly cytosolic in the *daf-2(e1370)*; *daf-16a*^*GR*^ strain, which shows the highest level of DAF-16a expression, while the *daf-2(e1370)*; *daf-16a::gfp*^*CF*^ and *daf-2(e1370)*; *daf-16a::gfp*^*TJ*^ strains showed an intermediate nuclear localization of DAF-16a. Importantly, since these different transgenic strains were generated with different methods and may be integrated at different locations, this indicates that it is the level of mRNA and protein of DAF-16a that determines its regulatory ability.

We reasoned that if a *daf-16a* transgene is expressed at close to endogenous levels, the *daf-16a* transgenic animals should not live longer than wild-type animals expressing endogenous levels of all three DAF-16 isoforms. Interestingly, our low-copy *daf-16a::gfp*^*HT*^ transgenic worms lived shorter than wild-type (Figure 2B, Additional file 1: Table S3), suggesting that low level expression of *daf-16a* alone cannot replace loss of the endogenous *daf-16* for lifespan regulation. However, the three high-copy *daf-16a* transgenic strains lived longer than wild type (Figure 2B, Additional file 1: Table S3). Therefore, these data suggest that the level of DAF-16a expressed from the *daf-16a::gfp*^*CF*^, *daf-16a::gfp*^*TJ*^, and *daf-16a::gfp*^*GR*^ transgenes exceeds the inhibitory capacity of the IIS pathway. This gene dosage effect was even more pronounced when each *daf-16a* transgene was analyzed in the *daf-16(mgDf50)*; *daf-2(e1370)* double mutant background (Figure 2C, Additional file 1: Table S3). As shown in Figure 2B and consistent with previous findings [19-21], when expressed at low levels from a *daf-16a::gfp*^*HT*^ transgene, DAF-16a cannot fully compensate for a complete loss of DAF-16 function (Figure 2B) [21]. An increase in lifespan was observed with increased expression of DAF-16a (*daf-16a::gfp*^*CF*^, *daf-16a::gfp*^*TJ*^). However, as the dosage of DAF-16a continued to increase (for example, *daf-16a::gfp*^*GR*^), a reduction in lifespan was seen (Figure 2C, Additional file 1: Table S3). Consistent with this finding, unlike the other *daf-16a* transgenic strains, the *daf-16a::gfp*^*GR*^ showed severely delayed development and reduced fecundity, suggesting that extremely high levels of DAF-16a are toxic (Additional file 1: Figure S3). Together, these findings suggest that the three commonly used strains for analyses of DAF-16:GFP do not accurately reflect the endogenous function of DAF-16a in lifespan regulation. Furthermore, these results indicate that low-copy *daf-16a* strains are more relevant for functional analyses as well as for DAF-16 localization assays (Additional file 1: Figures S2).

Next, we examined the low-copy *daf-16d/f::gfp*^*HT*^ transgene [21], which had approximately four-fold higher transcript level than endogenous *daf-16d/f* levels in the *daf-2(e1370)* mutant background (Additional file 1: Table S2). Unlike high-copy *daf-16a* and *daf-16d/f* transgenic worms [21], these *daf-16(mgDf50); daf-16d/f::gfp*^*HT*^ transgenic worms did not live longer than wild-type (Figure 2B, Additional file 1: Table S3), suggesting that DAF-16d/f activity in the low-copy *daf-16d/f* transgenic strain is regulated within the inhibitory capacity of the IIS pathway. Even though the strains were generated by different methods, we suggest that it is the level of the DAF-16 isoform protein that leads to an environment that does not accurately reflect endogenous levels of DAF-16.

### DAF-16d/f and DAF-16a respond differently to changes in IIS

**Figure 3:**
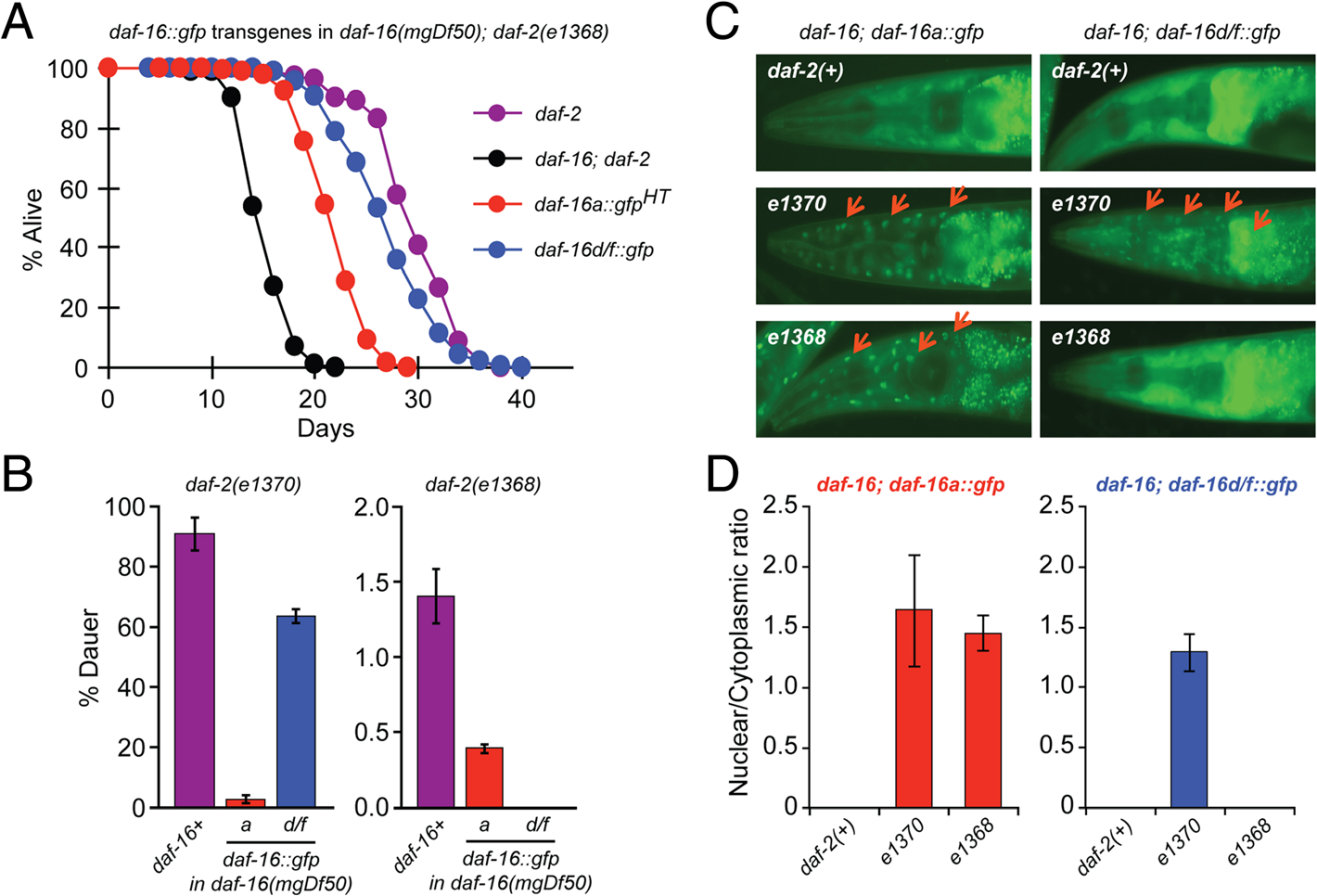
**DAF-16d/f and DAF-16a respond differently to changes in insulin/IGF-1 signaling. (A)** Lifespan analysis of *daf-16(mgDf50)*; *daf-2(e1368)* worms carrying *daf-16a::gfp*^*HT*^ or *daf-16d/f::gfp*^*HT*^. One of three repeats is shown; each with similar results. All of the lifespan data are shown in Additional file 1: Table S4. **(B)** Dauer formation in *daf-16(mgDf50)*; *daf-2(e1370)* or *daf-16(mgDf50)*; *daf-2(e1368)* worms carrying *daf-16a::gfp*^*HT *^*or daf-16d/f::gfp*^*HT*^ at 20°C. The *daf-16(mgDf50)*; *daf-2(e1368)*; *daf-16d/f* strain did not form dauers, while *daf-16(mgDf50)*; *daf-2(e1370)*; *daf-16d/f* strain formed a significant fraction of dauers. Dauer formation data represents one experiment with additional repeats showing similar results (Additional file 1: Figure S5). **(C)** Localization of DAF-16 isoforms in *daf-2*(*e1368)* and *daf-2(e1370)* mutant backgrounds. *daf-16(mgDf50)*; *daf-2(e1368)* or *daf-16(mgDf50)*; *daf-2(e1368)* worms carrying *daf-16a::gfp*^*HT *^*or daf-16d/f::gfp*^*HT*^, as well as the *daf-2* mutants expressing endogenous *daf-16* isoforms, were visualized after incubation at 20°C for 20 hours. Red arrows indicate nuclear DAF-16. **(D)** Quantification of the nuclear localization of the DAF-16 isoforms in *daf-2*(*e1368)* and *daf-2(e1370)* mutant backgrounds. Nuclear enrichment of DAF-16d/f is observed in *daf-2(e1370)* worms but not in *daf-2(e1368)* worms*,* while nuclear enrichment of DAF-16a is observed in both *daf-2* alleles.

The low-copy *daf-16* isoform transgenes were crossed into the background of another widely used *daf-2* allele, *daf-2(e1368)*, which exhibits weaker longevity and dauer arrest phenotypes than *daf-2(e1370)*[5,41]. Based on our lifespan data in the *daf-16(mgDf50)*; *daf-2(e1370)* background, we expected that the *daf-16d/f::gfp*^*HT*^ transgene would be sufficient to fully complement the lifespan of a *daf-16(mgDf50)*; *daf-2(e1368)* double mutant. Yet, to our surprise, *daf-16(mgDf50)*; *daf-2(e1368)*; *daf-16d/f::gfp*^*HT*^ transgenic worms lived significantly shorter than the *daf-2(e1368)* single mutant (Figure 3A, Additional file 1: Figure S4, Additional file 1: Table S3, Additional file 1: Table S4). In contrast, the *daf-16a::gfp*^*HT*^ transgene rescued lifespan to similar levels in both *daf-2(e1368)* and *daf-2(e1370)* mutant backgrounds (Additional file 1: Figure S4, Additional file 1: Table S3, Additional file 1: Table S4).

To further test the effect of DAF-16d/f in the *daf-2(e1370)* and *daf-2(e1368)* mutant backgrounds, we examined dauer formation as an additional output of DAF-16 activity. At the restrictive temperature of 25°C, both *daf-2(e1370)* and *daf-2(1368)* form 100% dauers whereas *daf-16(mgDf50)*; *daf-2(e1370)* and *daf-16(mgDf50)*; *daf-2(e1368)* double mutants do not form any dauers*.* However, once again, the DAF-16d/f transgene had different effects in the two *daf-2* alleles*: daf-16(mgDf50)*; *daf-2(e1368)*; *daf-16d/f::gfp*^*HT*^ transgenic worms formed approximately 39% dauers, whereas *daf-16(mgDf50)*; *daf-2(e1370)*; *daf-16d/f::gfp*^*HT*^ worms formed approximately 98% dauers at 25°C (Additional file 1: Figure S5A). At the semi-permissive temperature of 20°C, a similar trend was observed. This is in contrast to *daf-16a* transgenic worms where dauer formation was comparable in both *daf-2* mutant backgrounds (Figure 3B, Additional file 1: Figure S5).

Examining the DAF-16 nuclear/cytosolic localization, DAF-16d/f was enriched in the nucleus in the *daf-2(e1370)* background, but more cytosolic in the *daf-2(e1368)* background (Figure 3C,D). In contrast, DAF-16a was primarily enriched in the nucleus in both *daf-2(e1368)* and *daf-2(e1370)* backgrounds (Figure 3C,D). Thus, the DAF-16 localization data correlated with both functional outputs - lifespan and dauer formation. Whereas DAF-16a is activated to a similar level in both alleles, DAF-16d/f is less active in the *daf-2(e1368)* mutant than in the *daf-2(e1370)* mutant. Therefore, these data show that DAF-16a and DAF-16d/f exhibit different thresholds for inhibition by the upstream IIS pathway.

### Transcription of *daf-16d/f* is upregulated during aging

Although the spatial expression patterns of *daf-16* have been extensively investigated [19,20,39], the temporal expression of *daf-16* has not been studied. We monitored the expression of the *daf-16 isoform::gfp* strains throughout development and for the first few days of adulthood. Interestingly, as shown in Additional file 1: Figure S6, the GFP intensity of the *daf-16d/f::gfp* transgenic worms dramatically increased in the adult stage compared to larval stages.

**Figure 4:**
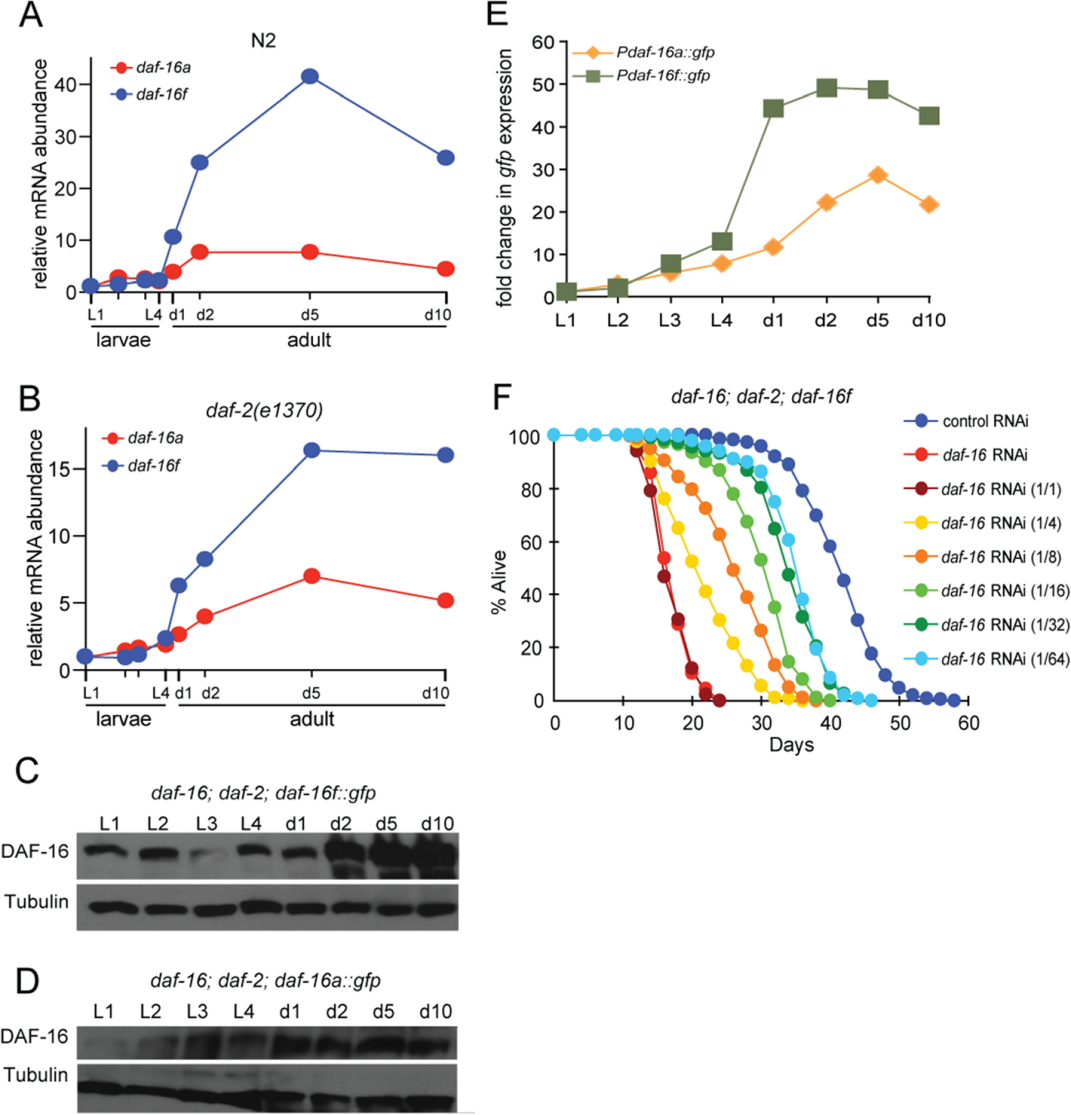
***daf-16d/f *is regulated at the level of transcription in both a temporal and spatial manner. (A,B)** Quantitative RT-PCR analysis of endogenous *daf-16a* and *daf-16d/f* mRNA levels during larval development (L1-L4) and in the adult. In both wild-type and *daf-2(e1370)* mutants, the level of *daf-16d/f* mRNA significantly increases with age. Additional details are shown in Additional file 1: Figure S7. **(C,D)** Changes in DAF-16d/f and DAF-16a protein levels with age. Equal volumes of worms were used for each larval stage. Western blots with the worm lysates were probed with anti-α-DAF-16 antibody (top panel) and anti-α-tubulin (bottom panel). DAF-16d/f shows a dramatic increase in protein levels with age as compared to DAF-16a. **(E)** Quantification of the fluorescence intensity in *Pdaf-16a::gfp* and *Pdaf-16d/f::gfp* transgenic worms as they age The GFP intensity was quantified using ImageJ software and normalized to the L1 stage for each strain. **(F)** Transcriptional regulation of *daf*-*16d/f* determines longevity. *daf-16(mgDf50)*; *daf-2(e1370)*; *daf-16d/f* transgenic worms were treated with diluted *daf-16* RNAi bacteria from the L4 stage to day 2 adulthood. All lifespan data are shown in Additional file 1: Table S5.

Next, we asked if the increase in GFP intensity in adult *daf-16d/f::gfp* transgenic animals correlates with the level of the endogenous transcript. Using qRT-PCR, we found that each of the *daf-16* isoforms was expressed at a constant level throughout larval development. However, as worms aged, the *daf-16d/f* transcript dramatically increased in both wild-type and long-lived *daf-2(e1370)* mutants, while the *daf-16a* transcript increased only slightly (Figure 4A,B, Additional file 1: Figure S7-S9). To further confirm this result, we tested the upregulation of DAF-16a vs DAF-16d/f at the protein level. Since it is not possible to distinguish differences in endogenous levels of DAF-16a and DAF-16d/f protein, we were limited to examining changes in DAF-16 protein levels in strains that only bear one DAF-16 isoform (*daf-2;daf-16;daf-16a::gfp* or *daf-2;daf-16;daf-16d/f::gfp*). Using these transgenic strains, consistent with our mRNA data, the age-dependent increase in the levels of DAF-16d/f was also observed at the protein level (Figure 4C,D).

The qRT-PCR assay is a read-out of the steady-state level of mRNA, but the DAF-16::GFP fusion fluorescence is the product of both transcriptional and post-transcriptional regulation. Therefore, to measure the transcriptional promoter activity of each *daf-16* isoform, we generated *daf-16* promoter::GFP fusions (*Pdaf-16::gfp*). The promoter of each *daf-16* isoform was placed upstream of the GFP open reading frame fused to the *unc-54* 3′UTR, which is known to be absent of temporal regulatory elements [42]. We reasoned that if increased transcription is responsible for the elevated mRNA level in young adults, then the GFP intensity should become brighter in the *promoter::gfp* transgenic worms as the worms age. In the *Pdaf-16a::gfp* worms, the GFP signal throughout the body increased slightly with age. In contrast, in *Pdaf-16d/f::gfp* worms, GFP expression increased dramatically throughout the intestine (data not shown Figure 4E). Therefore, across multiple independent experiments we found that transcription of *daf-16*, particularly *daf-16d/f*, was regulated in an age-dependent manner.

### *daf-16d/f* transcript level in the early adult determines lifespan

*daf-16* expression in the intestine is critical for lifespan regulation in *C. elegans*[21,43], and our temporal and spatial expression data indicate that *daf-16d/f* mRNA is dramatically upregulated in the intestine as worms age. To determine the importance of DAF-16 upregulation in aging worms, we used RNAi to maintain *daf-16d/f* expression in the adult stage at a level comparable to that in the L4 stage, thereby preventing the upregulation of DAF-16d/f during aging. To achieve the correct knockdown level, we prepared serial-dilutions of *daf-16* RNAi bacteria (from no dilution to 64-fold dilution) and fed *daf-16(mgDf50)*; *daf-2(e1370)*; *daf-16d/f::gfp*^*HT*^ worms from the L4 stage to day 2 adults. After 2 days of growth on the *daf-16* RNAi plates, worms were transferred to control RNAi plates. We found that worms grown on four-fold to 16-fold diluted *daf-16* RNAi bacteria maintained *daf-16d/f* expression at levels similar to that of the L4 worms (Additional file 1: Figure S10). Importantly, the lifespan of *daf-16(mgDf50)*; *daf-2(e1370)*; *daf-16d/f::gfp*^*HT*^ worms fed on the four-fold to 16-fold diluted *daf-16* RNAi bacteria was reduced as compared to animals grown on control bacteria (Figure 4F, Additional file 1: Table S5). Therefore, the upregulation of *daf-16d/f* gene expression at the young adult stage is an important determinant of *C. elegans* lifespan.

### The ELT-2 transcription factor promote longevity by regulating both *daf-16a* and *daf-16d/f* expression

**Figure 5:**
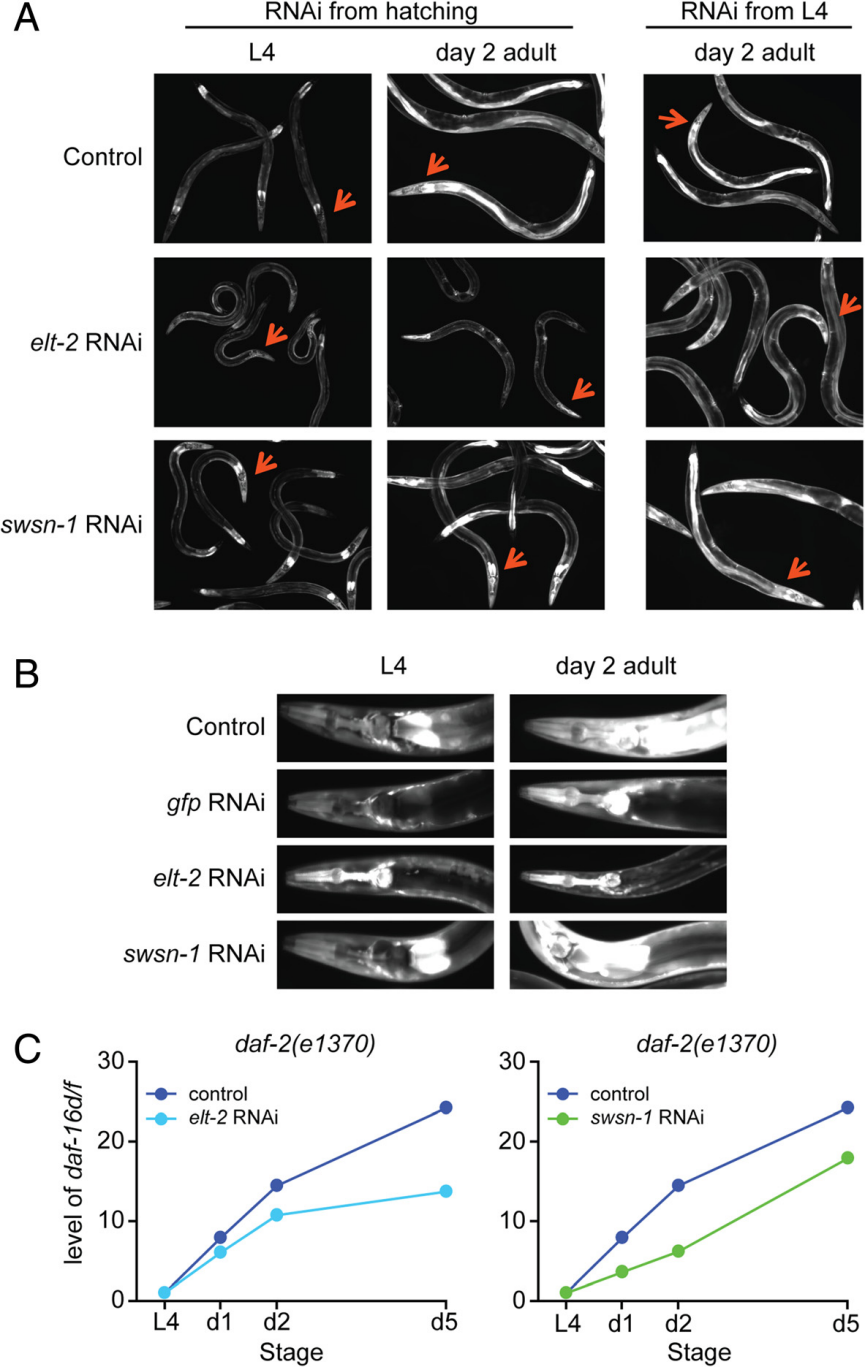
**ELT-2 and SWSN-1 are required for *daf-16 *gene expression. (A)***Pdaf-16d/f::gfp* expression grown on control, *elt-2* RNAi or *swsn-1* RNAi bacteria. Left and middle panels worms grown on RNAi from hatching, right panel worms grown on RNAi from L4. Red arrows indicate the head region of the worms. **(B)** Close-up of worms shown in Panel A Left and middle columns with additional GFP RNAi control. *Pdaf-16d/f::gfp* expression in the head and anterior intestine of worms grown on control, *gfp*, *elt-2*, or *swsn-1* RNAi bacteria from hatching. **(C)** Quantitative RT-PCR analysis of endogenous *daf-16d/f* in L4 larvae and Days 1, 2, and 5 adult *daf-2(e1370)* worms. For *elt-2*, worms were grown on RNAi bacteria from L4 stage and for *swsn-1* were grown on RNAi bacteria from hatching. The expression of *daf-16d/f* is reduced by *elt-2* or *swsn-1* RNAi treatment. Additional details are shown in Additional file 1: Figure S13.

Our findings suggest that transcriptional regulators expressed in the intestine activate *daf-16d/f* transcription in an age-dependent manner. To identify regulator(s) of *daf-16d/f* gene expression, we performed an RNAi screen of 892 transcriptional regulators using *Pdaf-16d/f::gfp* transgenic worms. RNAi of two transcriptional regulators reduced *Pdaf-16d/f::gfp* expression: *elt-2*, a GATA transcription factor; and *swsn-1*, a core component of the SWI/SNF chromatin remodeling complex (Figure 5A, B,C). *elt-2* is essential for intestinal development [31,32,44,45] as well as innate immune responses to bacterial and fungal infection [46] and directly regulates the expression of >80% of intestinal genes, including genes that are downstream of *daf-16* (*dod* gene) [32].

**Figure 6:**
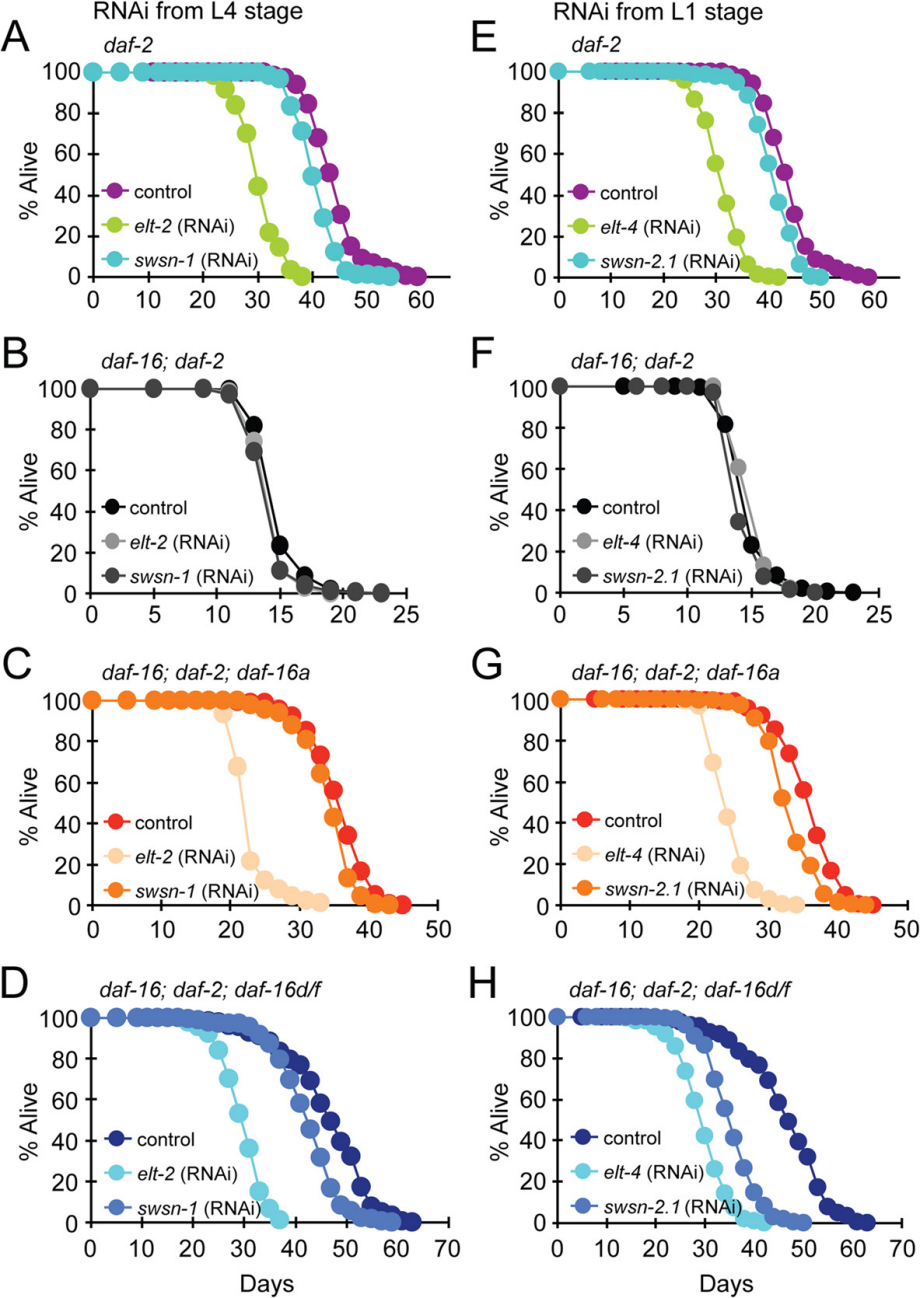
**ELT-2, ELT-4, and SWI/SNF promote longevity by regulating *daf-16 *gene expression. (A-D) ***elt-2* and *swsn-1* RNAi were started at the L4 stage. **(E-H) ***elt-4* and *swsn-2.1* RNAi were started at the L1 stage (hatching). Lifespan analyses of *daf-2(e1370)* (A, E), *daf-16(mgDf50)*; *daf-2(e1370)* (B, F), *daf-16(mgDf50)*; *daf-2(e1370)*; *daf-16a::gfp* (C, G), and *daf-16(mgDf50)*; *daf-2(e1370)*; *daf-16d/f::gfp* (D, H). One of three repeats is shown; each with similar results. All lifespan data are summarized in Additional file 1: Table S6.

Because worms fed with *elt-2* RNAi from hatching develop an abnormal intestinal structure and die within a few days, we used post-developmental RNAi to silence *elt-2* beginning at the L4 stage when intestinal development is complete. Indeed, post-developmental *elt-2* RNAi treatment also reduced *Pdaf-16d/f::gfp* expression (Figure 5B). When we examined the endogenous *daf-16* transcripts, we found that post-developmental *elt-2* RNAi treatment reduced the expression of endogenous *daf-16d/f* compared to control RNAi (Figure 5C). In contrast, the *Pdaf-16a::gfp* was only marginally downregulated on *elt-2* RNAi at day 2 and endogenous *daf-16a* expression was not changed, likely due to the fact that its intestinal expression is only a minor portion of total *daf-16a* (Additional file 1: Figure S11) [21]. Accordingly and consistent with a recent report [47], *elt-2* RNAi reduced the lifespan of the *daf-2(e1370)* single mutant (expressing endogenous *daf-16*), as well as *daf-16(mgDf50)*; *daf-2(e1370)*; *daf-16a::gfp*^*HT*^ and *daf-16(mgDf50)*; *daf-2(e1370)*; *daf-16d/f::gfp*^*HT*^ transgenic worms (Figure 6A, C, D, Additional file 1: Table S6). Relative to the control RNAi, *elt-2* RNAi did not shorten the lifespan of a *daf-16(mgDf50)*; *daf-2(e1370)* mutant (Figure 6B), suggesting that *elt-2* RNAi shortens lifespan by reducing intestinal *daf-16* gene expression and not by a *daf-16*-independent pathway.

We analyzed several additional GATA transcription factors for a role in *daf-16* transcriptional regulation and longevity, including *elt-4* and *elt-7*, which are expressed in the intestine [44], and *elt-3*, *elt-5*, and *elt-6*, which were previously implicated in lifespan regulation [48]. Only *elt-4* RNAi reduced the expression of the *Pdaf-16d/f::gfp* transcriptional reporter (Additional file 1: Figure S13)*,* though less dramatically than *elt-2* RNAi. Furthermore, only *elt-4* RNAi reduced the lifespan of both *daf-16(mgDf50)*; *daf-2(e1370)*; *daf-16a::gfp*^*HT*^ and *daf-16(mgDf50)*; *daf-2(e1370)*; *daf-16d/f::gfp*^*HT*^ transgenic worms (Figure 6E–H, Additional file 1: Table S6). Neither *Pdaf-16d/f::gfp* expression nor lifespan was affected by *elt-7*, *elt-3*, *elt-5*, or *elt-6* RNAi (Additional file 1: Figure S13 and Additional file 1: Table S6). These data are consistent with a recent study [49] suggesting that the previously reported role of ELT-3 in lifespan regulation [48] should be re-evaluated. Our findings indicate that the ELT-2 and ELT-4 GATA factors promote longevity by regulating intestinal *daf-16* expression.

### SWI/SNF determines lifespan by regulating *daf-16d/f* expression

The second positive clone we obtained from our screen was *swsn-1*, which encodes a core component of the highly conserved SWI/SNF nucleosome-remodeling complex [50]. In *C. elegans*, SWI/SNF is required for asymmetric cell division [51] and differentiation [52]. Prior to the L4 stage, *Pdaf-16d/f::gfp* expression was unaffected by *swsn-1* RNAi (Figure 5A,B). In adult worms, however, the upregulation of GFP in the mid-intestine is severely reduced by *swsn-1* RNAi (Figure 5B). Expression of endogenous *daf-16d/f* was also reduced in animals exposed to *swsn-1* RNAi (Figure 5C, Additional file 1: Figure S12B). Interestingly, *swsn-1* RNAi did not significantly change the expression of *Pdaf-16a::gfp* (Additional file 1: Figure S11C), suggesting that SWSN-1 regulates the temporal and spatial expression of *daf-16d/f*, specifically. RNAi targeting *snfc-5* and *swsn-2.1*, core and accessory subunits of SWI/SNF, respectively, also reduced the temporal upregulation of *daf-16d/f* expression with age (Additional file 1: Figure S12) but did not change *daf-16a* expression, indicating that the SWI/SNF complex specifically controls *daf-16d/f* expression.

We next asked if SWI/SNF plays a role in lifespan regulation. Both *swsn-1* and *snfc-5* are essential genes [51] and hatched larvae grown on *swsn-1* or *snfc-5* RNAi develop into sick adults with pleiotropic defects [51,53]. To avoid these complications in lifespan analysis, we used post-developmental (L4) RNAi to silence *swsn-1*. However, *Pdaf-16d/f::gfp* expression was largely unaffected by post-developmental *swsn-1* RNAi (data not shown) and we observed only a marginal reduction of lifespan in *daf-16(mgDf50)*; *daf-2(e1370)*; *daf-16d/f::gfp*^*HT*^ transgenic worms (Figure 6D, Additional file 1: Table S6), suggesting that post-developmental (L4) RNAi does not efficiently silence *swsn-1*. Worms exposed to *swsn-2.1* RNAi from hatching, however, were much healthier than animals grown on *swsn-1* or *snfc-5* RNAi, which allowed us to assess the effect of SWI/SNF on lifespan. As shown in Figure 6H and Additional file 1: Table S6, *swsn-2.1* RNAi shortened the lifespan of *daf-16(mgDf50)*; *daf-2(e1370)*; *daf-16d/f::gfp*^*HT*^ worms, but only modestly reduced the lifespan of *daf-2(e1370)* or *daf-16(mgDf50)*; *daf-2(e1370)*; *daf-16a::gfp*^*HT*^ animals (Figure 6F,G, Additional file 1: Table S6). Interestingly, a very recent paper found that SWI/SNF interacts with DAF-16 [54]. These studies revealed that a SWI/SNF-DAF-16 complex co-localizes at the promoters of DAF-16 direct targets and activates genes important for lifespan, dauer formation, and stress resistance [54]. Therefore, these results support a biochemical interaction of DAF-16 and SWI/SNF. Taken together, our data are consistent with a model in which the SWI/SNF complex directly interacts with DAF-16 to specifically promote longevity by regulating *daf-16d/f* expression.

## Conclusion

In *C. elegans*, the single FOXO family member, DAF-16 regulates lifespan, metabolism, development, and stress resistance by generating multiple isoforms including DAF-16d/f, DAF-16a, and DAF-16b [21]. Comparing the relative abundances of the different isoforms revealed that *daf-16d/f* is the most abundant isoform in early adulthood and that transcription of *daf-16d/f* mRNA is dramatically increased in the intestine as animals age. These findings are in agreement with previous studies showing that DAF-16 expression in the intestine is important for lifespan regulation and that lifespan is determined in adulthood [43,55]. Therefore, the temporal control of *daf-16d/f* transcription in the intestine is a critical determinant of longevity.

Importantly, temporal regulation of *FOXO* expression is conserved in mammals. In rats, *FOXO3* and *FOXO4* transcript are undetectable in very young animals but increase as animals age in the duodenum [56], and human *FOXO1* mRNA is significantly enriched in muscle samples from old individuals [57]. It will be particularly interesting to determine whether the age-dependent increase in mammalian FOXO expression are regulated at the level of transcription, as we have shown for *daf-16d/f* in *C. elegans*.

In worms, flies, and mammals, FOXO is the primary target of the IIS pathway [2,58]. In *C. elegans*, the DAF-2 IIS receptor has been extensively studied by genetic analysis. The *daf-2(e1370)* allele harbors a mutation in the tyrosine kinase domain, whereas *daf-2(e1368)* has a mutation in the ligand-binding domain [5]. These *daf-2* alleles show phenotypic differences, with *daf-2(e1370)* displaying longer lifespan and stronger dauer arrest compared to *daf-2(1368*) [41,58], indicating that the IIS pathway is more active in *daf-2(e1368)* than in *daf-2(e1370)*. Our lifespan, dauer, and DAF-16 nuclear localization data in these *daf-2* mutants revealed that *daf-16d/f* and *daf-16a* respond differently to changes in the levels of IIS. In both mutant backgrounds, DAF-16a is predominantly nuclear indicating that DAF-16a is activated to a similar level in both *daf-2* mutants. However, DAF-16d/f is nuclear only in the *daf-2(e1370)* background and therefore appears to be more active in this background. This suggests that a small decrease in IIS activates DAF-16a, whereas large decreases in IIS will activate DAF-16d/f and could add another layer of regulation by the different DAF-16 isoforms.

Using an RNAi screen, we identified the GATA factor ELT-2 and the SWI/SNF subunit SWSN-1 as factors required for *daf-16d/f* expression. Our functional analysis reveals that the intestinal GATA transcription factors, ELT-2 and ELT-4, regulate *daf-16* expression and, in turn, longevity. Our findings are consistent with the work of McGhee et al. [32], who identified multiple GATA binding sites upstream of both *daf-16a* and *daf-16d/f* and proposed that intestinal GATA factors, particularly ELT-2, might regulate the expression of *daf-16*. Moreover, the promoters of many DAF-16 targets also have GATA binding sites [32,48,59], suggesting that ELT-2/ELT-4 may cooperate with DAF-16 to regulate longevity genes in the intestine.

Recent studies suggest that epigenetic control of gene expression is important for the aging process [60]. In *C. elegans*, IIS-dependent and IIS-independent epigenetic modifications have been linked to longevity [61-64]. Our study shows that SWI/SNF regulates lifespan by promoting the age-dependent activity of a FOXO gene. Importantly, components of the SWI/SNF complex are required for the age-dependent upregulation of *daf-16d/f* but not *daf-16a*. SWSN-2.1 is homologous to mammalian BAF60, a subunit known to interact with a number of transcriptional activators [65,66]. BAF60, has three variants (a, b, c) [66] and BAF60c has been shown to interact with the GATA4 transcription factor to promote differentiation in early mouse heart development [67]. We envision a similar role in stimulation of *daf-16d/f* transcription in the adult intestine. For example, ELT-2/ELT-4 GATA or other transcription factor(s) together with SWSN-2.1/BAF60 may interact with the *daf-16d/f* promoter during development. At the young adult stage, an intestine-specific developmental cue may stimulate the SWSN-2.1-dependent recruitment of the core SWI/SNF machinery to remodel the *daf-16d/f* promoter and activate *daf-16d/f* transcription.

**Figure 7:**
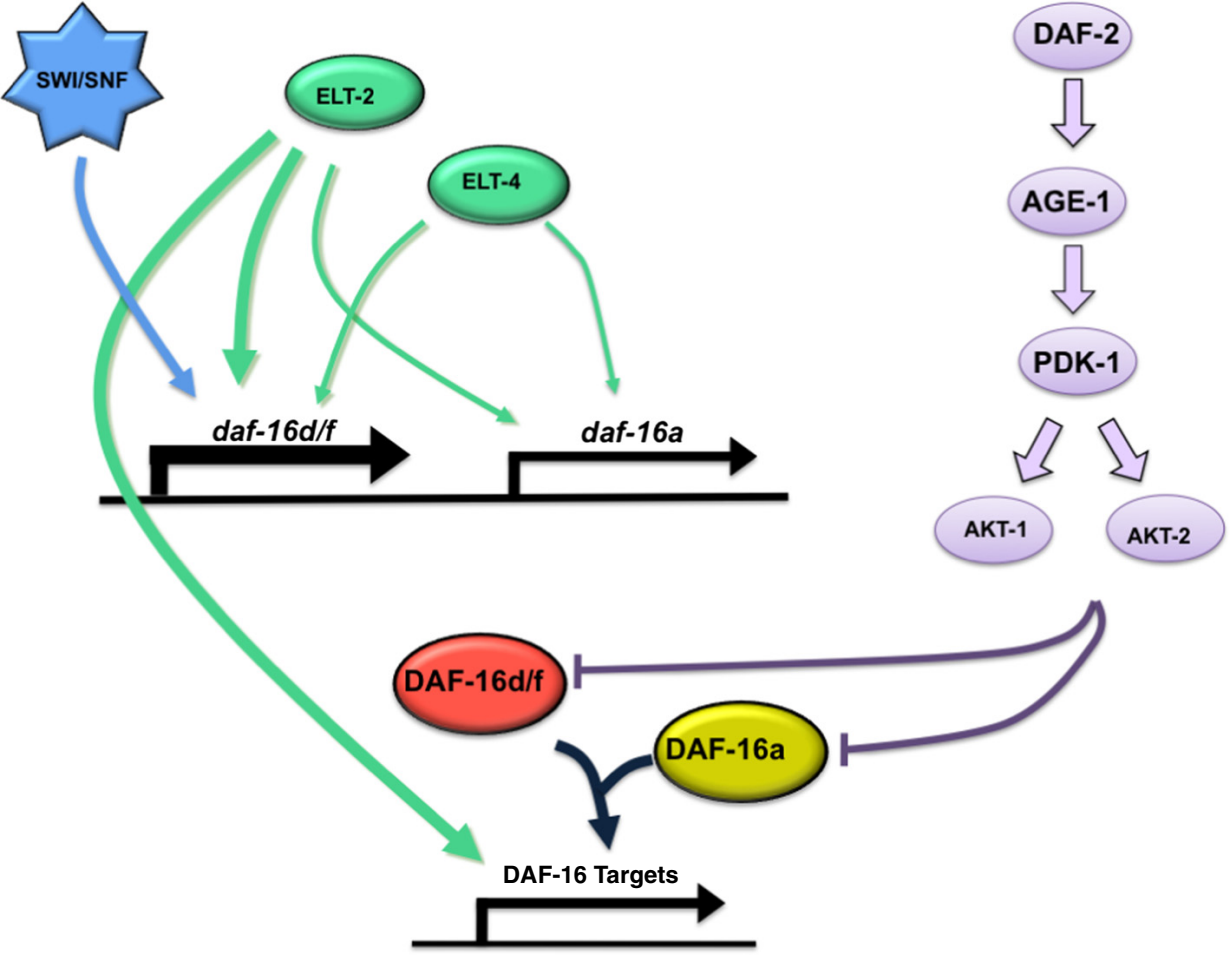
**Model of the transcriptional regulation of *daf-16. ****daf-16d/f* plays a more prominent role than *daf-16a* in lifespan regulation. The thickness of the line represents the strength of regulation. ELT-2 and ELT-4 are shown as direct regulators of DAF-16. SWI/SNF is shown as the direct regulator of DAF-16d/f and not DAF-16a.

We have shown here and elsewhere [21] that *daf-16a* and *daf-16d/f* are sensitive to changes in gene dosage. For *daf-16d/f*, mRNA and protein are dramatically upregulated with age, whereas *daf-16a* mRNA only minimally changes with age, and preventing the upregulation of *daf-16d/f* in adults shortens lifespan. ELT-2 and ELT-4 are likely to be direct regulators of DAF-16. Indeed, multiple GATA binding sites exist upstream of both *daf-16a* and *daf-16d/f* contain (data not shown). Based on our finding that SWI/SNF specifically regulates the expression of *daf-16d/f* but not *daf-16a*, we propose that SWI/SNF modifies the activity of the *daf-16d/f* promoter but not the *daf-16a* promoter. Taken together, and consistent with our findings, we suggest a model where *daf-16d/f* plays a more prominent role in lifespan regulation than *daf-16a* (Figure 7).

*C. elegans* has proved to be an invaluable model system for understanding the regulation and biological functions of FOXO transcription factors. The mechanisms that control FOXO proteins at a post-translational level are remarkably conserved from worms to mammals. Our findings indicate that transcriptional control of *daf-16/FOXO* is also an essential regulatory event. As there is increasing evidence that FOXOs are required for human longevity [68-73], our findings will advance our understanding of the regulation and function of DAF-16/mammalian FOXO in aging and age-dependent diseases including cancer and diabetes.

## Methods

### Strain maintenance

All strains were maintained and handled as described [74]. Animals were grown on standard NGM plates at 15°C using standard *C. elegans* techniques, unless otherwise indicated. Mutants used in this study included, LGI: *daf-16(mgDf50*); LGIII: *daf-2(e1368, e1370)*; *unc-119(ed3).* All transgenic worms have an *unc-119(ed3)* mutation rescued by an *unc-119*^*+*^ co-transformation marker. Transgenic strains generated and/or used in this study are listed in Additional file 1: Table S8.

### Strain construction

i) ***daf-2(e1368) unc-119(ed3) *double mutants**

Both genes map to Chromosome II but are 13 map units apart. *daf-2(e1368)* males were mated to *unc-119(ed3)* hermaphrodites. Approximately 20 F1 progeny were picked onto individual plates and allowed to produce progeny at 25°C. F2 *daf-2(e1368)* dauers were selected from each plate and allowed to recover at 15°C. From the F3 progeny, Unc worms were selected, generating *daf-2(e1368)*; *unc-119(ed3)*.

ii) ***daf-16(mgDf50)*; *daf-2(e1368) unc-119(ed3) *mutants**

*daf-16(mgDf50)*; *daf-2(e1368)* males were mated to *daf-2(e1368)*; *unc-119(ed3)* hermaphrodites. Approximately 20 F1 progeny were picked onto individual plates and allowed to have progeny at 25°C. From the F2 progeny on each plate, Unc Non-dauer worms were selected and tested for the *daf-16(mgDf50)* mutation by PCR. Primer sequences are listed in Additional file 1: Table S7. *daf-16(mgDf50)*; *daf-2(e1370)*; *unc-119(ed3)* mutants were used for microparticle bombardment [75] to generate *daf-16* isoform transgenic worms.

### DNA construction for *daf-16* isoform specific GFP and RNAi

To generate *daf-16 isoform::gfp* constructs, each cDNA encoding *daf-16a*, *daf-16d/f* was cloned into the pGEM-T vector. The clones were then verified by sequencing. For the *daf-*16a isoform promoter construct, a *Sal*I/*Bam*HI fragment containing 6.0 kb upstream of *daf-16a* was cloned into pPD95.75 (GFP containing plasmid). For the *daf-16d/f* isoform promoter construct, a *Sph*I/*Bam*HI fragment containing 4.1 kb upstream of *daf-16d/f* was cloned into pPD95.75 (GFP containing plasmid). We then generated full-length *daf-16a1* cDNAs using mutagenic primers with *Bam*HI/*Sma*I restriction enzyme sites, which were subcloned into pPD95.75 with the upstream promoter fragment. For *daf-16d/f*, since we were unable to amplify the *daf-16d/f* specific cDNA, we generated a construct which could generate both DAF-16d1 and DAF-16d/f by subcloning the 4.1 kb upstream genomic DNA next to the 5’ putative start codon of *daf-16d1* cDNA in frame [21].

To allow the constructs to be compatible with ballistic transformation, the *unc-119*^*+*^ gene was introduced into each vector using PCR redirected DNA recombination method [76]. To generate *daf-16* isoform specific RNAi vector, *Nhe*I/*Hin*dIII fragments covering isoform specific/overlapping cDNA were cloned into *L4440* vector [21]. The primers used for the PCR analysis are listed in Additional file 1: Table S7.

### RNA isolation and real-time PCR

#### Growing samples

To compare the expression of *daf-16* isoforms in developmental stages, worms were synchronized by bleaching, followed by tranferring eggs on plates seeded with OP50 bacteria. Worms were grown until the L1, L2, L3, and L4 stage and then harvested. To obtain aged worms, L4 stage worms were transferred to FuDR plates with final concentration of 0.1 mg/mL [77] seeded with OP50 bacteria. On days 1, 2, 5, and 10, worms were harvested and frozen at -80°C. To compare the expression of *daf-16a* in L4 stage, in Figure 2, worms were synchronized by bleaching, followed by L1 arrest in M9 buffer. The following day, the L1 worms were placed on plates seeded with OP50 bacteria and further incubated at 15°C until worms reached the L4 stage.

#### RNA preparation

Total RNA was isolated using acidic phenol (Sigma). Briefly, worms were washed off the plates using ice cold M9 buffer, followed by three additional washes. Next, 0.5 mL of AE buffer (acetic acid, EDTA), 0.1 mL of 10% SDS, and 0.5 mL of phenol were added and the mixture and vortexed vigorously for 1 min followed by incubation at 65°C for 4 min. The RNA was then purified by phenol:chloroform extraction followed by ethanol precipitation. The concentration and the purity of the RNA were determined by measuring the absorbance at 260/280 nm. To further determine the quality of the RNA, both the ribosomal 28 S and 18 S were visually inspected on an agarose gel. For Figure 4A and B, total RNA was purified from approximately 100 worms using Direct-zol^TM^ RNA MiniPrep kit (Zymo Research), as described by the manufacturer. cDNA was synthesized using total RNA and the SuperScript cDNA synthesis kit (Invitrogen, USA). Gene expression levels were then determined by real time PCR using the PowerSYBR® Green PCR Master Mix and StepOnePlus Real-Time PCR System (Applied Biosystems, USA). Relative gene expression was compared to actin as an internal control. Primers used are listed in Additional file 1: Table S7.

### Western blots

All strains were allowed to grow at 15°C. For Additional file 1: Figure S1, approximately 100 L4 stage worms were collected for each of the transgenic strain in 10 to 15 mL of M9 buffer. Worms were then washed once with M9. An equal amount of SDS containing loading buffer was added to the worm pellets and samples were immediately boiled to lyse the worms. Samples were cooled, centrifuged briefly, and the supernatant was loaded onto the gel. For the western blot analyses in Figure 4C and D, equal amounts (10 mL) of worms were used. For each experiment, the lysates were resolved on a 10% polyacrylamide gel by SDS-PAGE. Proteins were transferred to a nitrocellulose membrane and probed with antibody against DAF-16 [78] (1:2,500 dilution). The membrane was then reprobed with anti -α-tubulin (1:8,000 dilution).

### Growth assays

All the strains were grown at 15°C and five L4s were picked onto plates at 15°C. The plates were left undisturbed for 7 days and photos of each of the plates were taken using Nikon Coolpix995 camera to compare the growth of the strains.

### Lifespan assays

All lifespan analyses were performed at 20°C. Strains were semi-synchronized by allowing gravid adults to lay eggs overnight and then removing the adult worms. Worms were grown for several days until they reached the young adult stage at 15°C. Approximately 150 young adult worms were transferred to five freshly seeded plates containing FuDR to a final concentration of 0.1 mg/mL [77]. Worms were then scored as dead or alive by tapping them with a platinum wire every 2 to 3 days. Worms that died from vulval bursting were censored. Day 1 of the lifespan was when worms were transferred to the FuDR plate. Statistical analyses were done using the standard chi-squared-based log rank test. Lifespans were also verified on non-FuDR NGM plates (Additional file 1: Figure S15).

### DAF-16::GFP analysis

For measuring the nuclear:cytosolic ratio (Additional file 1: Figure S2 and Figure 3), worms were grown at 15°C until they reached young adult stage. Then, worms were mounted on glass slides in 50 mM sodium azide and visualized using Zeiss Axioscope 2+ microscope. Fluorescent images were taken using OpenLab.3.1.7 software with a Hamamatsu camera. For the quantification, only the region above intestinal cells in the pharynx was considered. Using ImageJ software, the fluorescence was quantified by measuring the pixel intensity in the nuclear *versus* cytosolic region. The ratio between the respective intensities was then calculated and plotted. This was repeated twice with approximately 14 to 15 worms in each assay.

### RNAi screen

RNAi knockdown by feeding was performed essentially as described in Timmons et al. [79] using a library of clones representing 892 of the predicted 937 *C. elegans* transcription factors (MacNeil and Walhout, unpublished). Briefly, 50 μL of an overnight culture of HT115 bacteria carrying RNAi clones was used to inoculate 1 mL LB supplemented with 50 μg/mL ampicillin. Cultures were grown, with shaking, for 6 h at 37°C. Bacteria were pelleted and resuspended in 10% of the original volume. NGM plates containing 5 mM IPTG were seeded with concentrated bacteria and allowed to dry. Synchronized L1 larvae were used to seed prepared RNAi plates. Animals were visually examined for changes in GFP expression during the adult stage.

## Availability of supporting data

The datasets- supporting figures for the results of this article are included as additional files.

## Competing interests

The authors declare that they have no competing interests.

## Authors’ contributions

AB contributed to the conception and design, data collection and analysis, manuscript writing, critical revision, and final approval of the manuscript. ESK contributed to the concept and design, data collection and analysis, manuscript writing, and final approval of the manuscript. DC contributed to manuscript writing, data analysis, and final approval of the manuscript. HL, MJG, and LTM contributed to data collection and final approval of the manuscript. HAT contributed to the conception and design, financial support, manuscript writing, and final approval of manuscript. All authors read and approved the final manuscript.

## Supplementary Material

Additional file 1 Supplemental Text. Click here for file
