# Supplementary material for: Transcriptional regulation of Caenorhabditis elegans FOXO/DAF-16 modulates lifespan
Source: Longev Healthspan. 2014 Apr 23;3:5. doi: 10.1186/2046-2395-3-5 (PMC4022319; doi:10.1186/2046-2395-3-5)
Supplement: Additional file 1 — Supplemental Text. [file 2046-2395-3-5-S1.pdf]

Supplemental Text

Transcriptional regulation of *Caenorhabditis elegans* FOXO/DAF-16 modulates lifespan

Ankita Bansal, Eun-Soo Kwon, Darryl Conte Jr , Haibo Liu, Michael J. Gilchrist, Lesley T. MacNeil, and Heidi A. Tissenbaum

Supplementary Data

***daf-16d/f* is the most abundant *daf-16* isoform in adult worms**

We find that *daf-16* is regulated at the level of transcription as worms age. To systematically analyze the comparative level of each *daf-16* isoform in the early adult stage, we designed isoform-specific primers as well as primers covering 3' sequences shared by each isoform (Table S7). Isoform-specific primers were specific to their respective target (data not shown). Since it is possible that the different *daf-16* primer sets could show different amplification efficiency, we first measured the *daf-16* mRNA level of *daf-16* isoform transgenic worms using isoform-specific primers as well as isoform-common primers (Figure S8A, B). Since these worms express the isoform specific transgene, the Ct values should be the same between the common and isoform specific values. However, we found that the isoform-specific primers and isoform-common primers produce different Ct values, Ct_{iso} vs Ct_{com} respectively, and this was most apparent for *daf-16b* (Figure S8-S9). Therefore, to directly compare the levels between the different *daf-16* isoforms, we calculated the ΔCt by subtracting the Ct_{com} value from the Ct_{iso} value (S10C). This is the $\Delta Ct_{calibration}$ (ΔCt_{S8}) shown in Figure S9. Importantly, for each *daf-16* isoform, the two biological repeats provided similar $\Delta Ct_{calibration}$ (ΔCt_{S8}) (Ct_{iso} - Ct_{com}) values (Figure S8C).

To compare the level of each *daf-16* isoform in day 1 adult *daf-2(e1370)* mutants, we first measured Ct value for each *daf-16* isoform using isoform-specific primers (Figure S9A); $Ct_{iso}^{daf\text{-}16a}$, $Ct_{iso}^{daf\text{-}16b}$, $Ct_{iso}^{daf\text{-}16d/f}$. Then, using the common primers, we measured the Ct value to obtain the $Ct_{com}^{daf\text{-}16\ total}$. The ΔCt for each isoform was determined by $Ct_{isoform} - Ct_{endogenous\ control}$ (Figure S9). Then to determine the ΔΔCt, we took the $\Delta Ct_{isoform} - \Delta Ct_{calibrator(from\ S8)}$ as shown in Figure S9. To determine the relative abundance of each *daf-16* isoform in day 1 adult *daf-2* mutants, we calculated $2^{-\Delta\Delta Ct}$ (Figure S9). Importantly, the sum of the relative levels of each *daf-16* isoform was around 1 (Figure S9B). Last, in two independent sample repeats, *daf-16d/f* was found to comprise more than 80 % of total *daf-16*, suggesting DAF-16d/f is the major isoform in early adult worms (Figure S9C).

Supplementary Figures:

Figure S1. Comparative DAF-16 protein levels in different *daf-16a* transgenic worm strains.

Equal amounts of lysates prepared from approximately 100 worms were loaded onto 10%SDS PAGE gel, and immunoblotted with anti-α-DAF-16 and anti-α-tubulin. See Methods for additional details.

Figure S2. Effect of dosage of transgene on the spatial distribution of DAF-16a

Nuclear/cytosolic distribution of DAF-16a in four *daf-16(mgDf50); daf-2(e1370); daf-16a* transgenic worms grown at 15°C. The nuclear/cytosolic ratio of DAF-16a::GFP was measured using Image J software. The mean ratio is plotted and the error bars represent the Standard Deviation. Two independent repeats are shown.

Figure S3. High doses of *daf-16a* transgene is toxic to worm

Comparison of the growth rates of different *daf-16a::gfp* transgenic strains in *daf-16(mgDf50)* background and *daf-16(mgDf50);daf-2(e1370*).

Figure S4. *daf-16a* and *daf-16d/f* regulate lifespan differently in response to levels of IIS knockdown

Lifespan analysis of *daf-16a* and *daf-16d/f* transgenes in either in *daf-2(e1370)*, and *daf-2(e1368)* genetic background. Bar graphs represent an average of the mean lifespan with the Standard Deviation for three independent experiments. All lifespan data are summarized in Table S3 and Table S4.

Figure S5. ***daf-16a*** **and** ***daf-16d/f*** **regulate dauer development differently in response to levels of IIS knockdown**

Dauer formation of *daf-16(mgDf50); daf-16* isoform transgenic worms in either a *daf-2(e1370)* or a *daf-2(e1368)* mutant background at 20°C and 25°C. (A) Repeat 1; At 20°C, mean dauer formation is as follows: *daf-2(e1370)*, 90.9 ± 5.5 % (n=440); *daf-16(mgDf50); daf-2(e1370)*, 0 % (n>300); *daf-16(mgDf50); daf-2(e1370); daf-16a*, 2.8 ± 1.4 % (n=787); *daf-16(mgDf50); daf-2(e1370); daf-16d/f*, 63.4 ± 2.4 % (n=719); *daf-2(e1368)*, 1.4 ± 0.2 % (n=785); *daf-16(mgDf50); daf-2(e1368)*, 0 % (n>300); *daf-16(mgDf50); daf-2(e1368); daf-16a*, 0.4 ± 0.0 % (n=775); *daf-16(mgDf50); daf-2(e1368); daf-16d/f*, 0 % (n=768). At 25°C, mean dauer formation is as follows: *daf-2(e1370)*, 100 % (n=498); *daf-16(mgDf50); daf-2(e1370)*, 0 % (n>300); *daf-16(mgDf50); daf-2(e1370); daf-16a*, 100 % (n=679); *daf-16(mgDf50); daf-2(e1370); daf-16d/f*, 100 % (n=662); *daf-2(e1368)*, 100 % (n=613); *daf-16(mgDf50); daf-2(e1368)*, 0 % (n>205); *daf-16(mgDf50); daf-2(e1368); daf-16a*, 97.9 ± 1.6 % (n=786); *daf-16(mgDf50); daf-2(e1368); daf-16d/f*, 38.9 ± 7.6 % (n=682). (B) Repeat 2; At 20°C, mean dauer formation is as follows: *daf-2(e1370)*, 98.5 ± 0.6 % (n=405); *daf-16(mgDf50); daf-2(e1370)*, 0 % (n=532); *daf-16(mgDf50); daf-2(e1370); daf-16a*, 13.9 ± 5.0 % (n=517); *daf-16(mgDf50); daf-2(e1370); daf-16d/f*, 64.2 ± 8.5 % (n=595); *daf-2(e1368)*, 8.8 ± 1.9 % (n=524); *daf-16(mgDf50); daf-2(e1368)*, 0 % (n=542); *daf-16(mgDf50); daf-2(e1368); daf-16a*, 2.6 ± 1.2 % (n=622); *daf-16(mgDf50); daf-2(e1368); daf-16d/f*, 0 % (n=554). At 25°C, mean dauer formation is as follows: *daf-2(e1370)*, 100 % (n=248); *daf-16(mgDf50); daf-2(e1370)*, 0 % (n>300); *daf-16(mgDf50); daf-2(e1370); daf-16a*, 100 % (n=548); *daf-16(mgDf50); daf-2(e1370); daf-16d/f*, 100 % (n=535); *daf-2(e1368)*, 100 % (n=402); *daf-16(mgDf50); daf-2(e1368)*, 0 % (n=205); *daf-16(mgDf50); daf-2(e1368); daf-16a*, 100 % (n=569); *daf-16(mgDf50); daf-2(e1368); daf-16d/f*, 80.2 ± 1.7 % (n=465).

Figure S6. Upregulation of *daf-16d/f* levels as the worms age

Temporal expression pattern of *daf-16d/f.* GFP become brighter with age in *daf-16(mgDf50); daf-2(e1370); daf-16d/f::gfp* transgenic worms. Worms were grown at 15°C and L2, L4, and day 2 worms were visualized with a 1 sec exposure time.

Figure S7. Endogenous transcript levels of *daf-16d/f* is upregulated with age

The expression profiles of three *daf-16* isoforms in wild type N2 (A, C) and long-lived *daf-2(e1370)* strains (B, D). The mRNA levels of *daf-16a*, *daf-16b*, *daf-16d/f* were determined using Quantitative-RT PCR analysis. In both repeats, the expression of *daf-16d/f* is upregulated, compared to *daf-16a* and *daf-16b*. The primer sequences are listed in Table S7.

Figure S8. Quantitative-RT PCR showing differential amplification efficiency with different primers.

(A, B) The mRNA levels of the *daf-16* isoforms in *daf-16(mgDf50); daf-2(e1370); daf-16 isoform* transgenic strains were measured using isoform specific primers and isoform common primers. (C) Respective Ct values (Ct_{iso}, and Ct_{com}) were calculated with threshold of 0.2. Two biological repeats provide similar ΔCt values (Ct_{iso} - Ct_{com}); $\Delta Ct^{daf\text{-}16a}$, 1.28 ± 0.06 ; $\Delta Ct^{daf\text{-}16b}$, 3.70 ± 0.29; $\Delta Ct^{daf\text{-}16d/f}$, 0.68 ± 0.04. $\Delta Ct_{calibration}$ values were used in the analysis of the relative abundance of each isoform in the transcriptional analysis of *daf-2(e1370)* in Figure S11.

Figure S9. *daf-16d/f* is the most abundant transcript in day 1 adult *daf-2(e1370)* worms.

(A) The mRNA levels of three *daf-16* isoforms of *daf-2(e1370)* worms were measured using isoform specific primers and isoform common primers. (B) The respective Ct values ($Ct_{iso}^{daf\text{-}16a}$, $Ct_{iso}^{daf\text{-}16b}$, $Ct_{iso}^{daf\text{-}16d/f}$, and $Ct_{com}^{daf\text{-}16total}$) were calculated with a threshold of 0.2. (C) Comparative mRNA levels of different *daf-16* isoforms. See Supplementary Text for Experimental Details.

Figure S10. Temporal *daf-16* RNAi treatment to *daf-16(mgDf50); daf-2(e1370); daf-16f::gfp* transgenic worms.

Transgenic worms were grown on HT115 bacterial food. L4 staged worms were transferred to serially diluted *daf-16* RNAi plates containing FUDR; (A) control food, (B) *daf-16* RNAi food, (C) 1/4 diluted *daf-16* RNAi food, (D) 1/16 diluted *daf-16* RNAi food, (E) 1/64 diluted *daf-16* RNAi food. After two days on RNAi plates, worms were moved back to control food. Pictures were taken at both 10X and 40X magnification as shown in the figure. Worms were visualized at indicated time points with 500mS exposure time. The ¼ and 1/16 dilutions maintained the GFP levels of day-2 old worms similar to L4 stages and were used for lifespan analysis.

Figure S11. Effect of *elt-2* and *swsn-1* RNAi on *Pdaf-16a::gfp* expression.

Pdaf-16a::gfp strains were grown on RNAi of *elt-2* or *swsn-1*. L4 stage or day 2 adult worms were visualized. The anterior part of the intestine was marked with a dotted line.

Figure S12. Transcription factors regulate the expression of *daf-16a* and *daf-16d/f*.

The temporal expression profiles of *daf-16a* and *daf-16d/f* in wild type or *daf-2(e1370)* strains when (A) *elt-2* or (B) *swsn-1* were knocked-down. The mRNA levels of *daf-16a* and *daf-16d/f* were determined using Quantitative-RT PCR analysis. In two biological repeats, the temporal upregulation of *daf-16d/f* is significantly reduced compared to control.

Figure S13. Effect by RNAi of GATA transcription factors on *Pdaf-16d/f::gfp* expression.

(A)Visualization of L4 stage *Pdaf-16d/f::gfp* transgenic worms. (B) Visualization of Day 2 adult *Pdaf-16d/f::gfp* worms. Worms are grown on the indicated RNAi bacteria from hatching.

Figure S14. Effect by RNAi of SWI/SNF complex components on *Pdaf-16d/f::gfp* expression.

(A) Visualization of L4 stage *Pdaf-16d/f::gfp* transgenic worms. (B) Visualization of Day 2 adult *Pdaf-16d/f::gfp* worms. Worms are grown on indicated RNAi bacteria from hatching.

Figure S15: Lack of an effect of FuDR on lifespan.

The strains show similar lifespan curves without FuDR. A concentration of 400uM FuDR was used.

Supplementary Tables:

Table S1- Analysis of the ESTs that correspond to *daf-16*

Table S2- Comparison of levels of *daf-16* isoform transcript

Table S3- Lifespan analysis of *daf-16* isoform transgenic strains

Table S4- Lifespan analysis of *daf-16* isoform transgenic strains

Table S5- Lifespan analysis of serially diluted *daf-16* RNAi

Table S6- Lifespan analysis of GATA transcription factors and SWI/SNF components

Table S7- List of Primers used in this study

Table S8- List of Strains used in this study

Figure S1. Comparative DAF-16 protein levels in different *daf-16a* transgenic worm strains

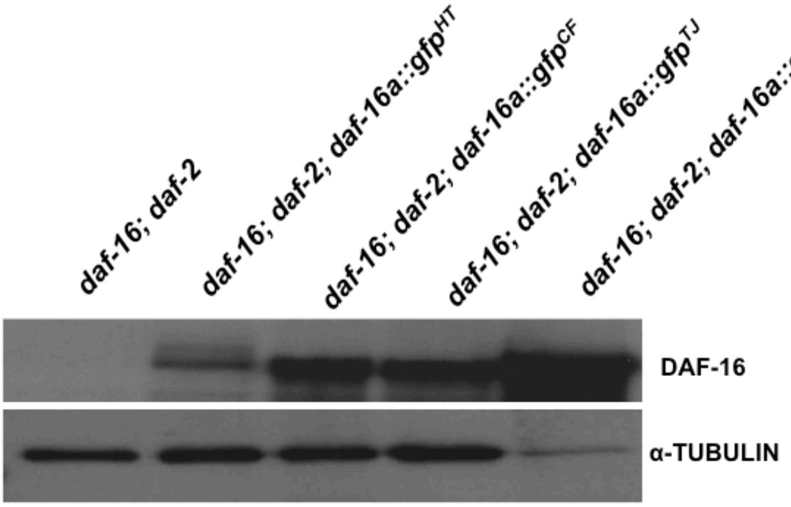

Figure S2. Effect of dosage of transgene on the spatial distribution of DAF-16a

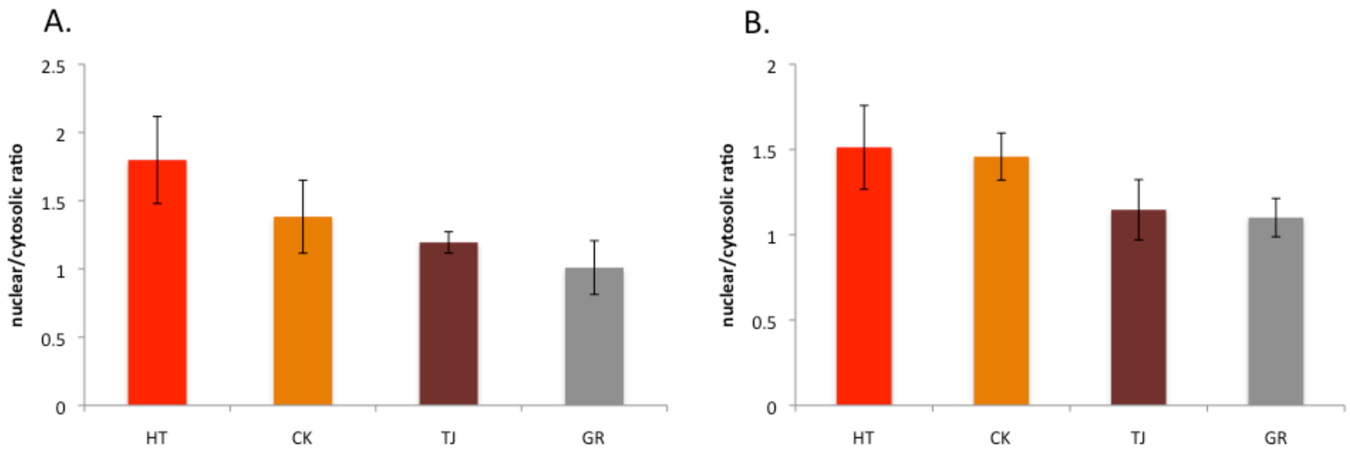

Figure S3. High doses of *daf-16a* transgene is toxic to worm

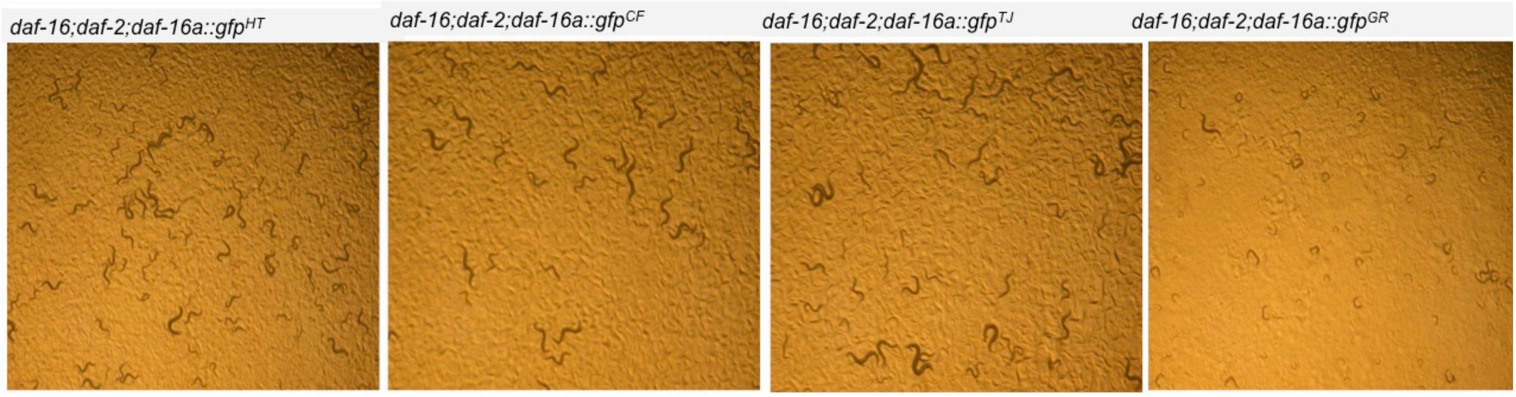

Figure S4. ***daf-16a*** **and** ***daf-16d/f*** **regulate lifespan differently in response to levels of IIS knockdown**

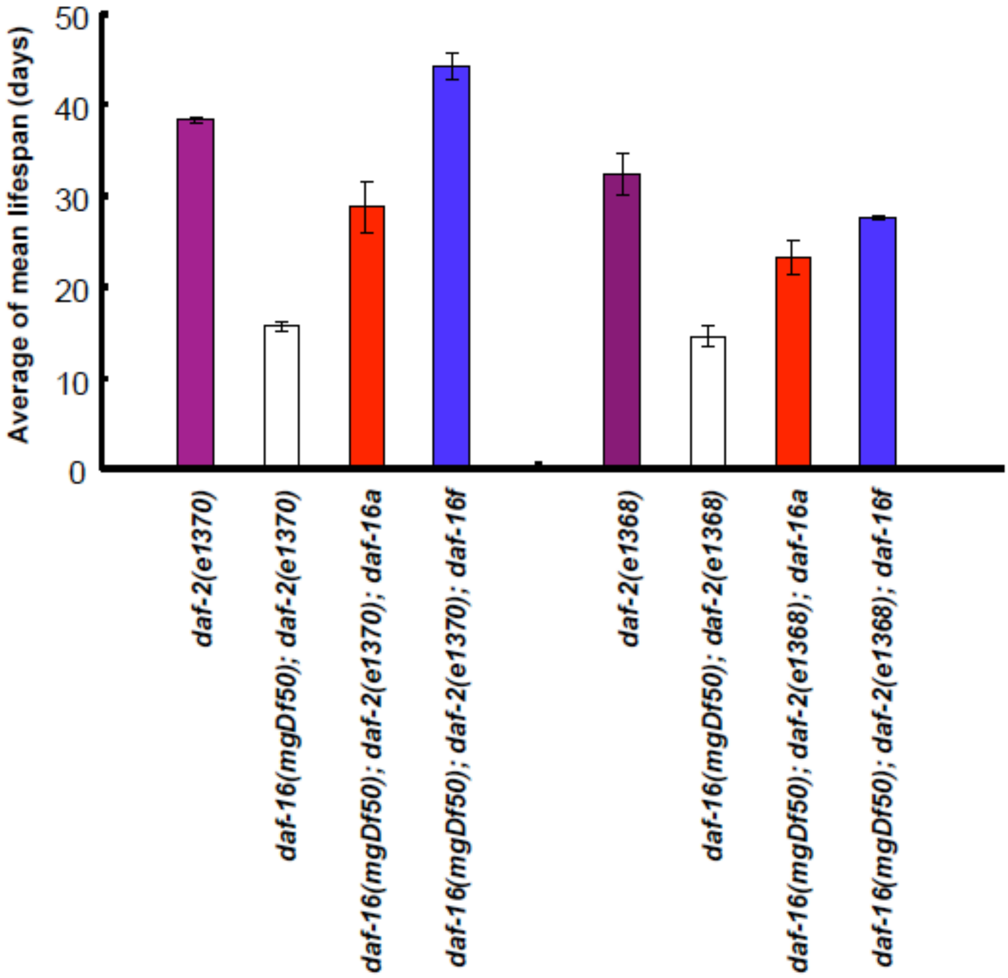

Figure S5. ***daf-16a*** **and** ***daf-16d/f*** **regulate dauer development differently in response to levels of IIS knockdown**

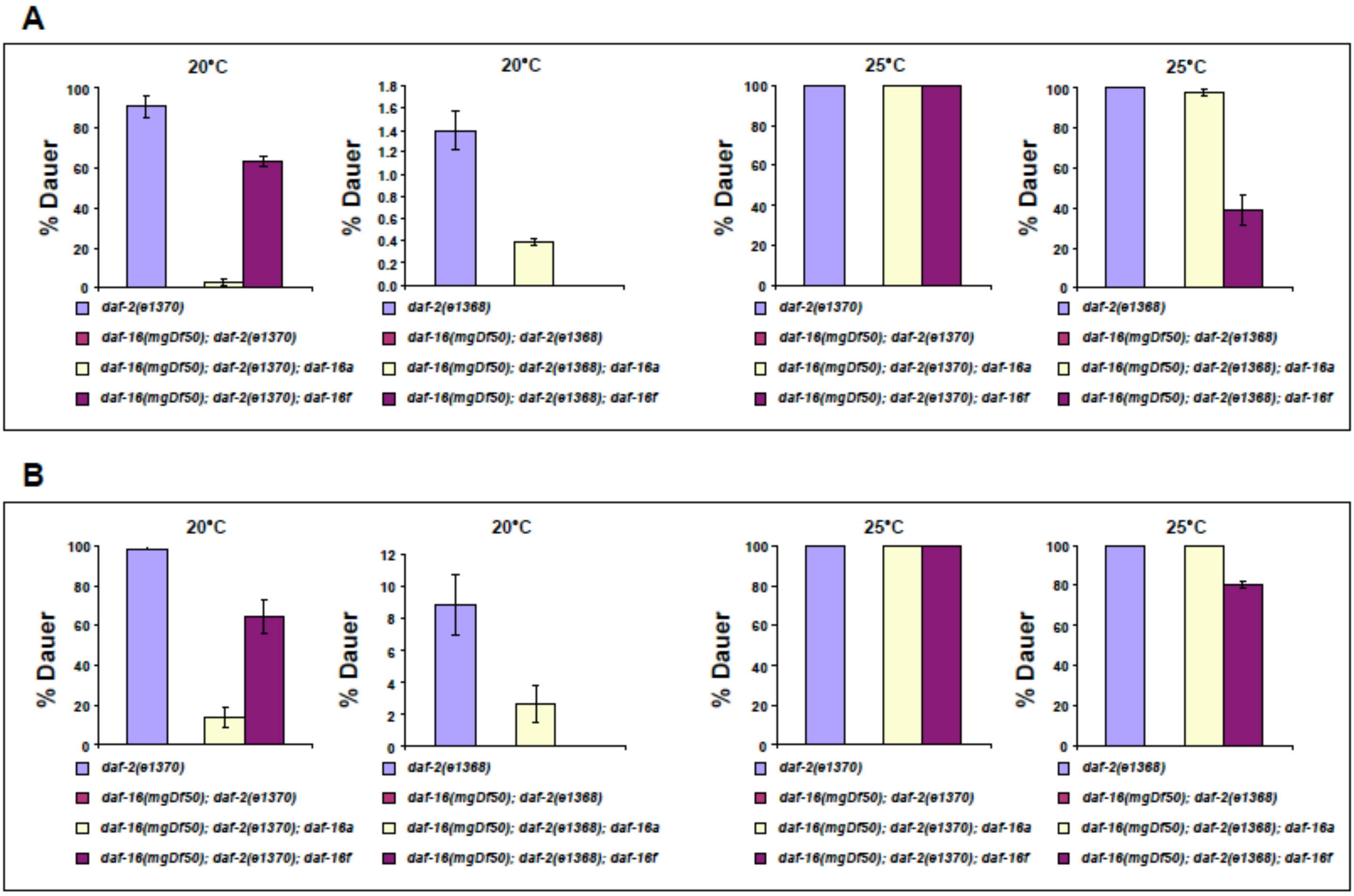

Figure S6. Upregulation of *daf-16d/f* levels as the worms age

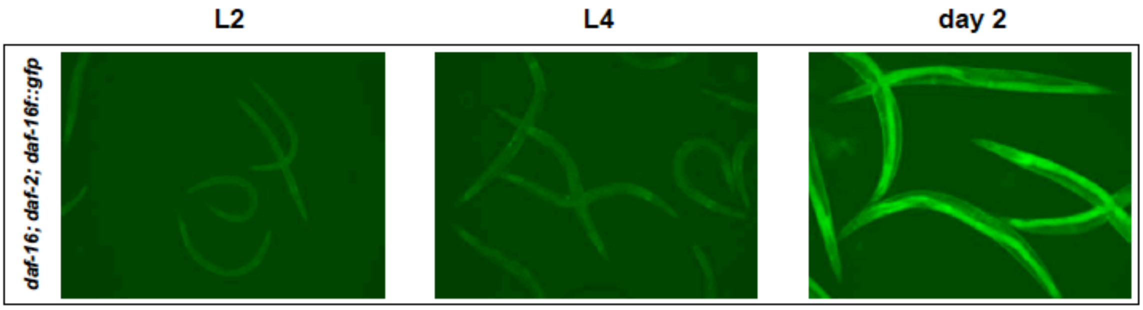

Figure S7. Endogenous transcript levels of *daf-16d/f* is upregulated with age

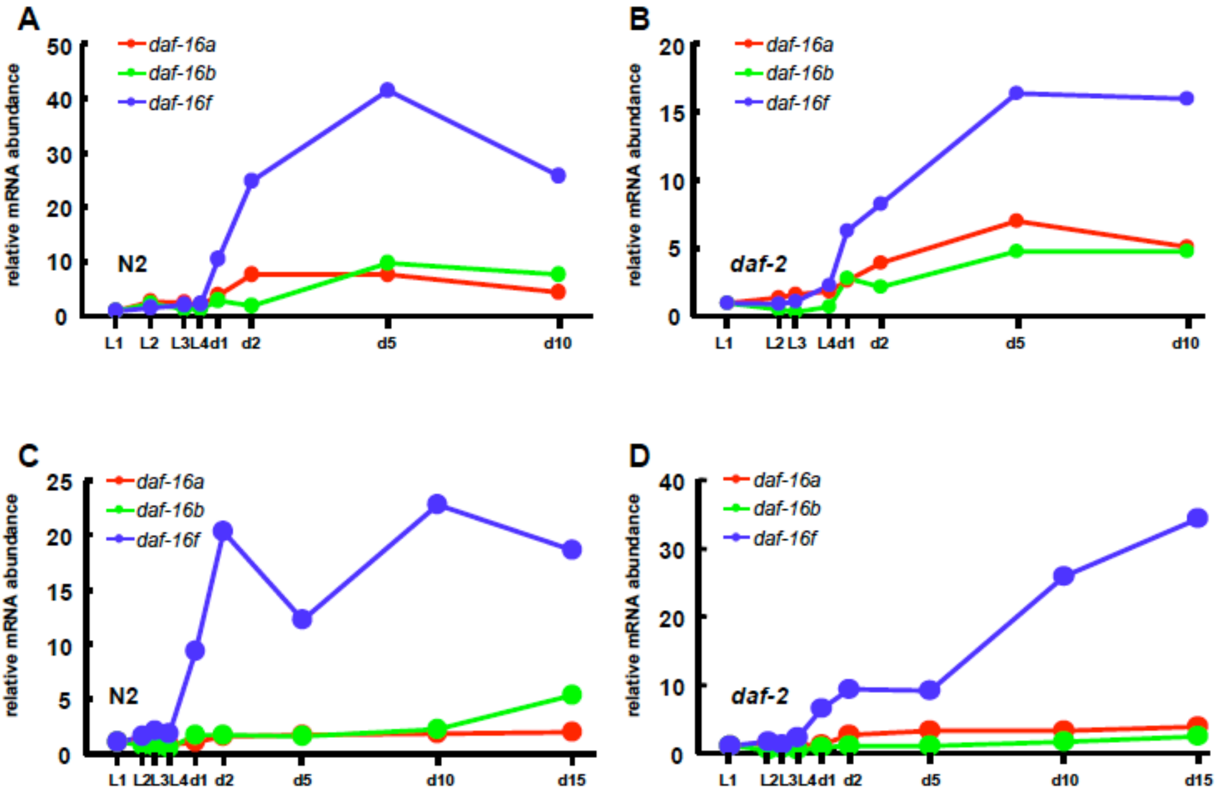

Figure S8. Quantitative-RT PCR showing differential amplification efficiency with different primers.

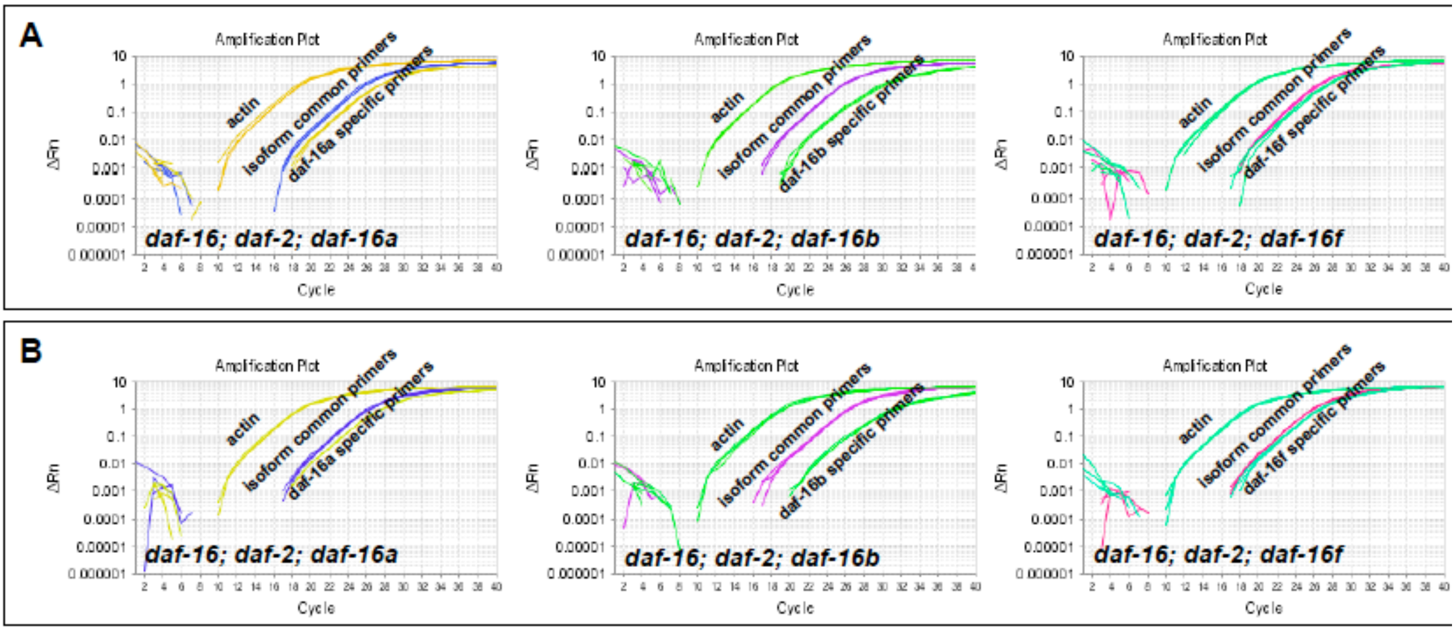

C

repeat 1 / repeat 2	Ct_{iso}	Ct_{com}	ΔCt ($Ct_{iso} - Ct_{com}$)
daf-16a	24.48 / 24.81	23.24 / 23.48	1.23 / 1.32
daf-16b	26.61 / 27.55	23.11 / 23.66	3.49 / 3.90
daf-16f	24.63 / 23.79	23.93 / 23.14	0.71 / 0.65

Figure S9. ***daf-16d/f*** **is the most abundant transcript in day 1 adult** ***daf-2(e1370)*** **worms.**

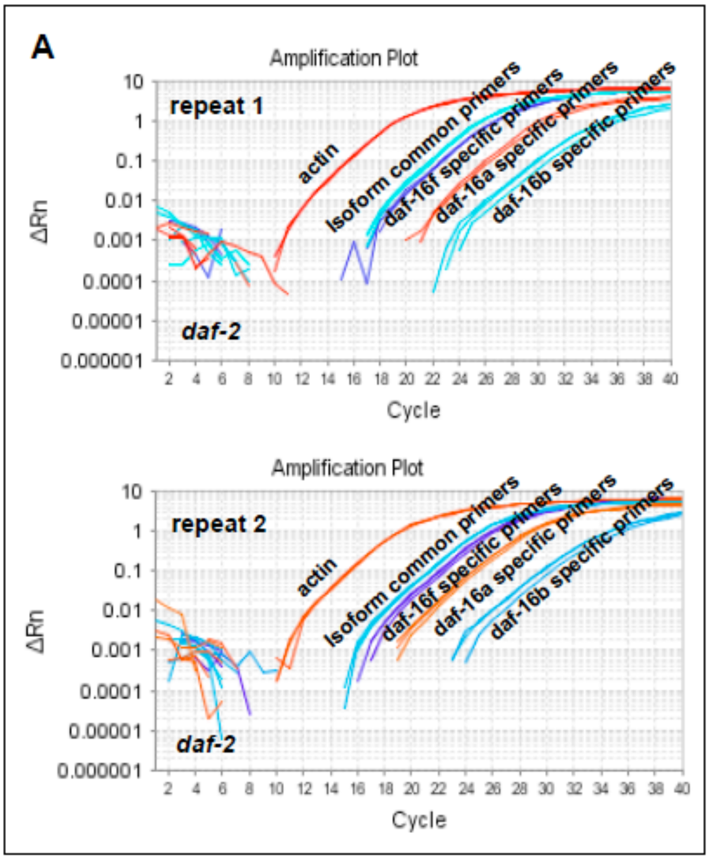

B

repeat 1	Ct_{iso}	Ct_{com}** (Ct_{iso}-ΔCt*)	Ct_{com}** – $Ct_{com}^{daf\text{-}16total}$	Relative abundance
daf-16a	27.30	26.06	3.21	0.11
daf-16b	31.11	27.62	4.76	0.04
daf-16f	23.76	23.06	0.20	0.87
daf-16total	N/A	22.86	0.00	1 (1.02)
**repeat 2**	Ct_{iso}	Ct_{com}** (Ct_{iso}-ΔCt*)	Ct_{com}** – $Ct_{com}^{daf\text{-}16total}$	Relative abundance
daf-16a	25.79	24.46	2.09	0.23
daf-16b	30.91	27.01	4.64	0.04
daf-16f	23.22	22.57	0.20	0.87
daf-16total	N/A	22.37	0.00	1 (1.14)

Figure S10A. ***daf-16(mgDf50); daf-2(e1370); daf-16f::gfp*** **transgenic worms grown on control food (vector RNAi)**

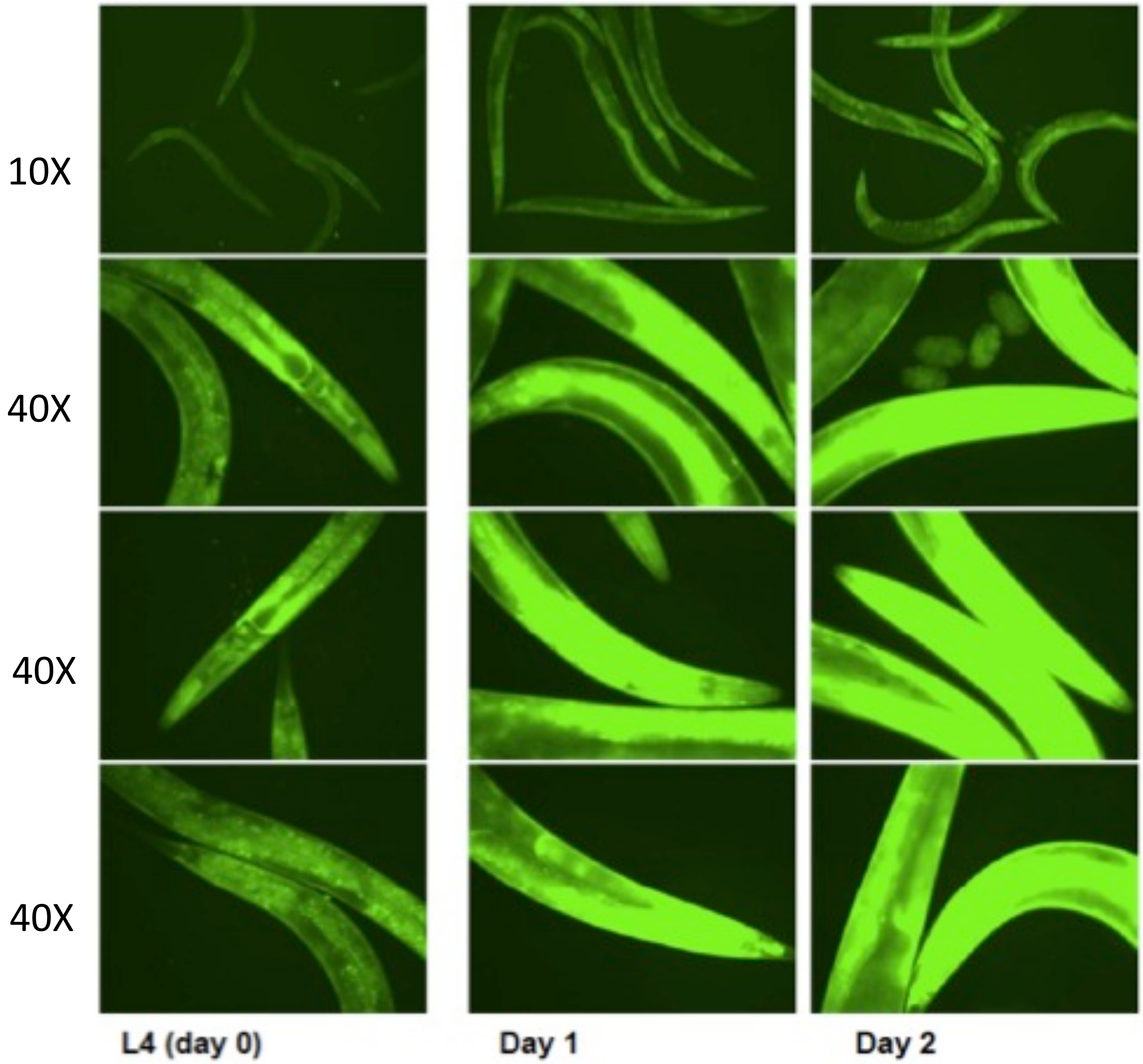

Figure S10B-*daf-16(mgDf50); daf-2(e1370); daf-16f::gfp* transgenic worms grown on *daf-16* RNAi food

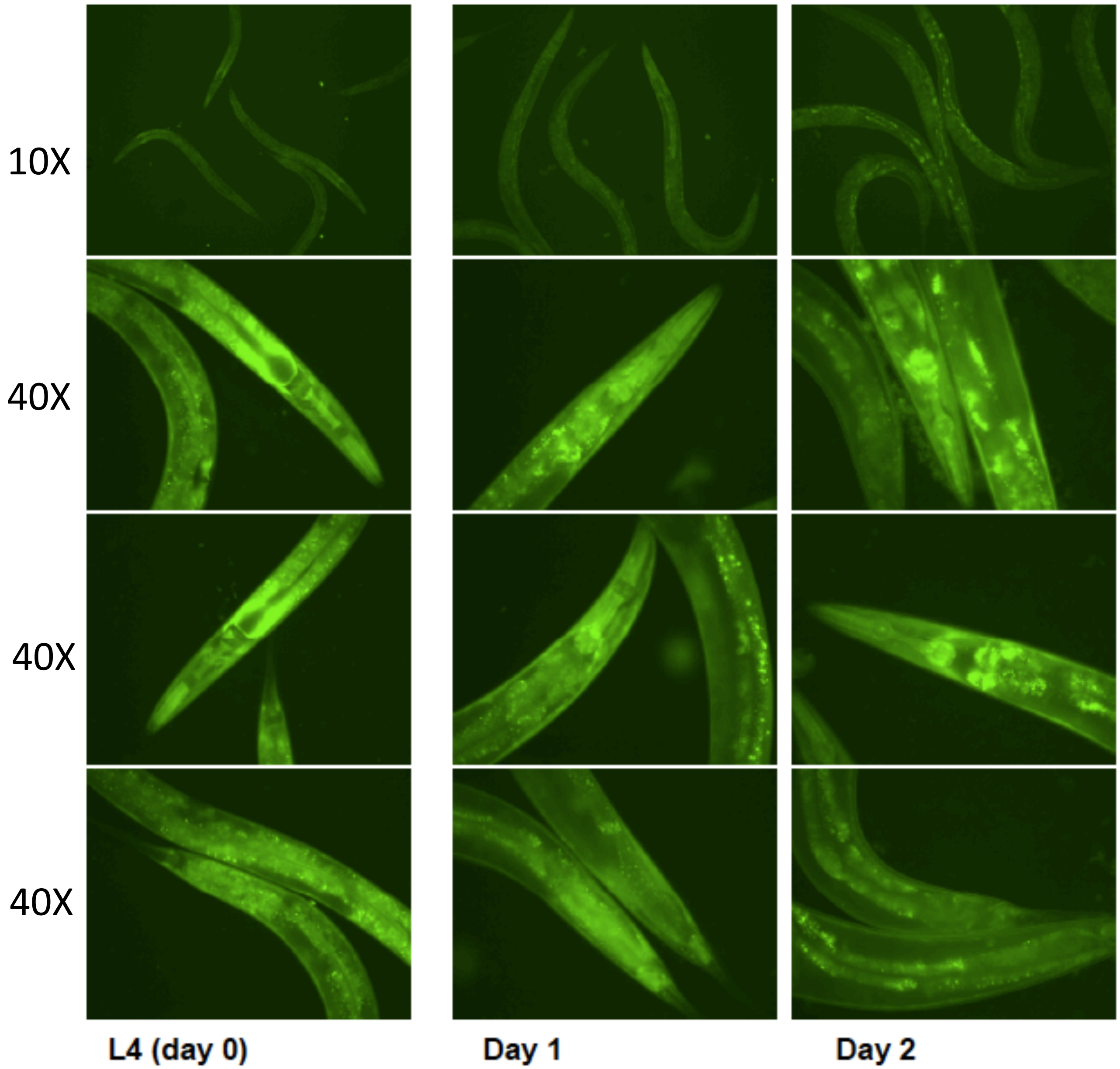

Figure S10C. ***daf-16(mgDf50); daf-2(e1370); daf-16f::gfp*** **transgenic worms grown on 1/4 diluted *daf-16* RNAi food**

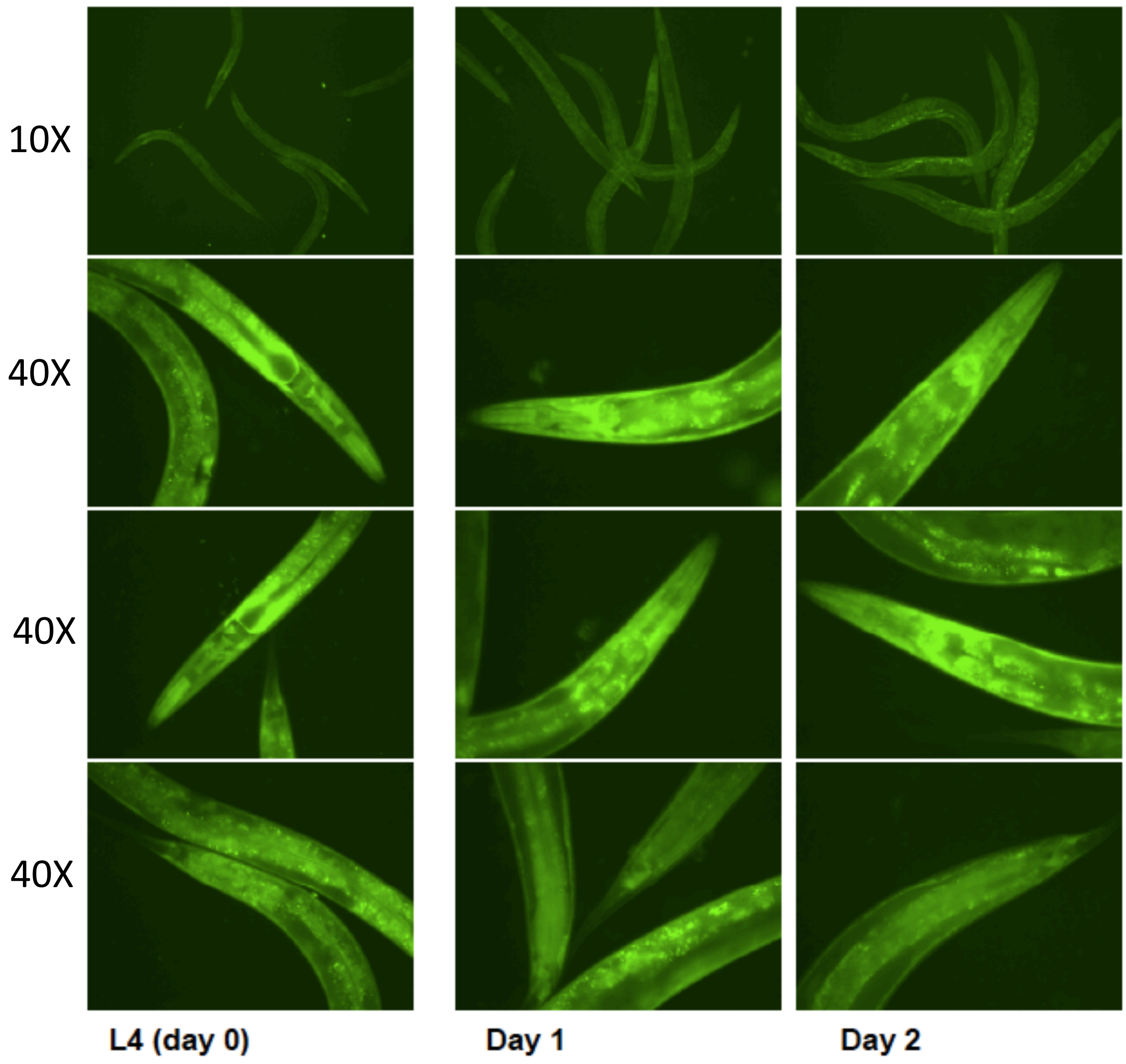

Figure S10D. *daf-16(mgDf50); daf-2(e1370); daf-16f::gfp* **transgenic worms grown on 1/16 diluted** *daf-16* **RNAi food**

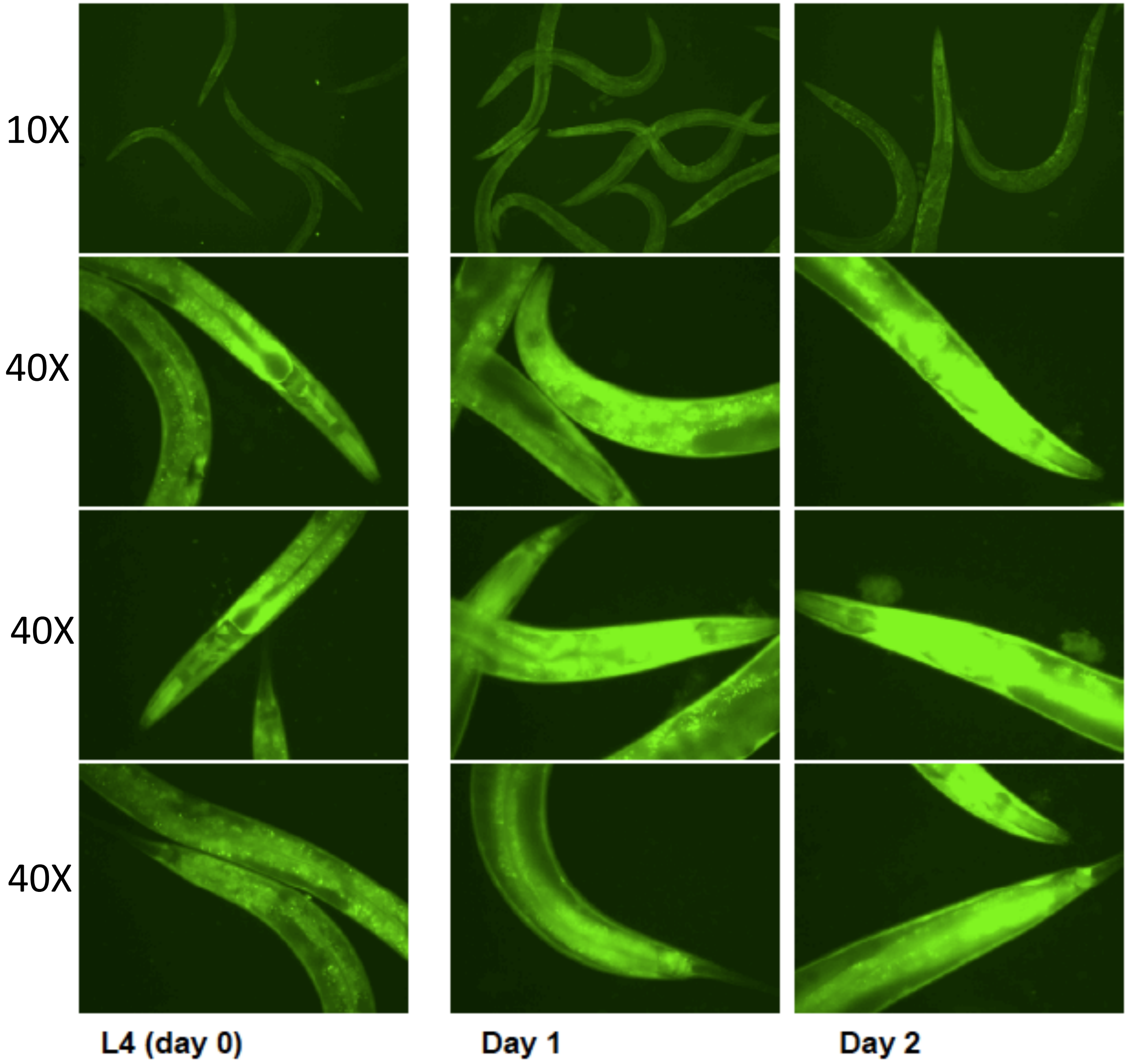

Figure S10E. ***daf-16(mgDf50); daf-2(e1370); daf-16f::gfp*** **transgenic worms grown on 1/64 diluted** ***daf-16*** **RNAi food**

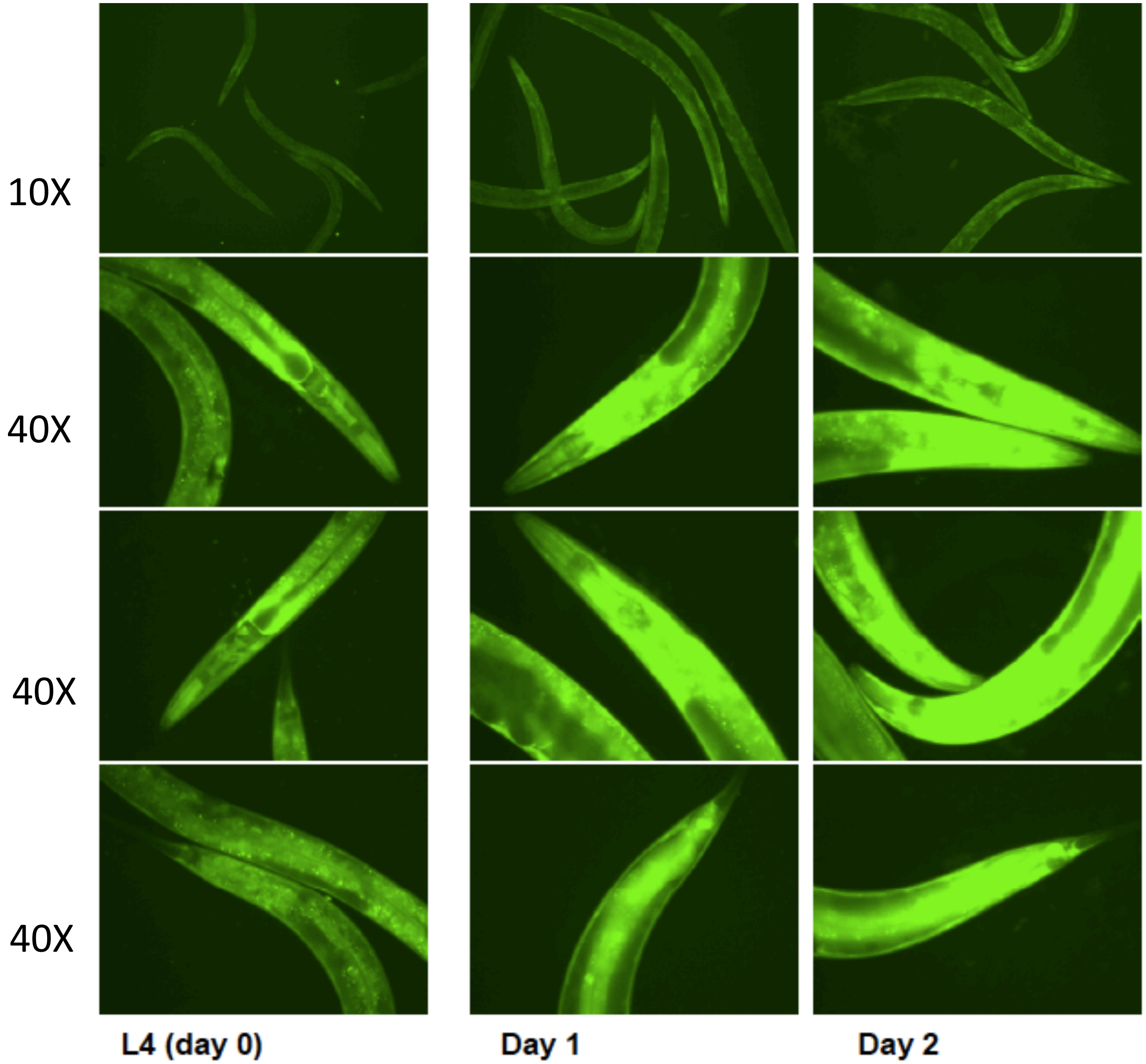

Figure S11. Effect of *elt-2* and *swsn-1* RNAi on *Pdaf-16a::gfp* expression.

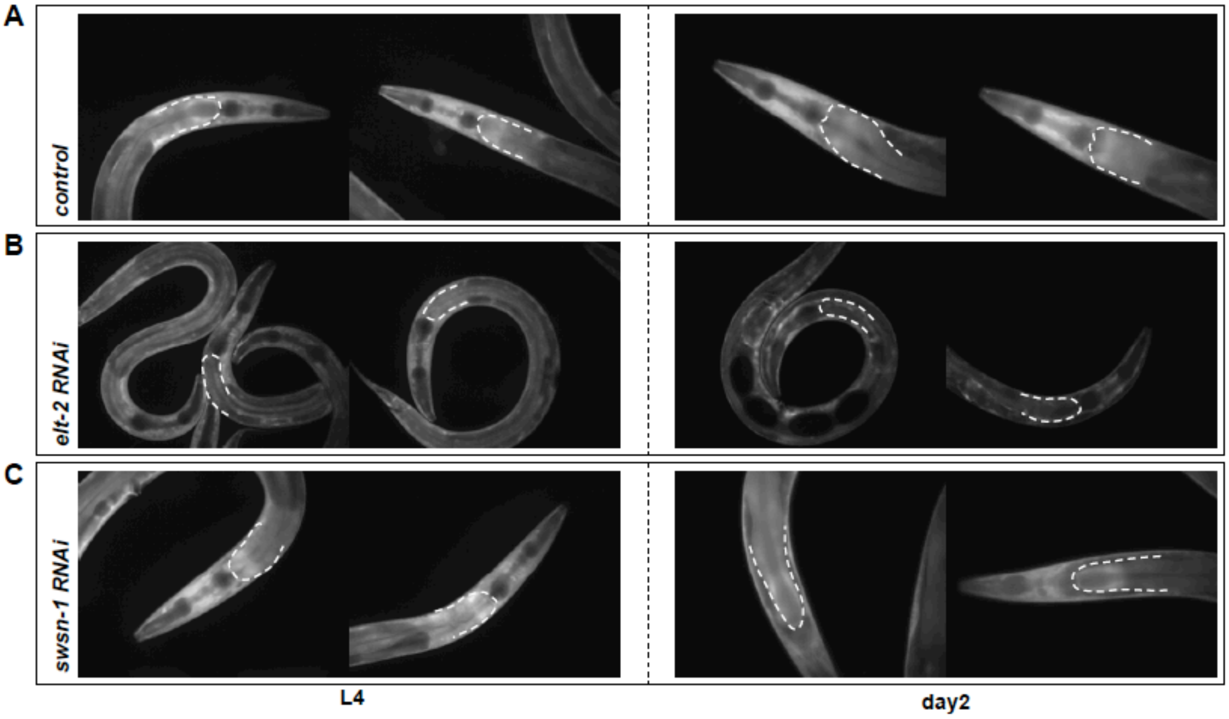

Figure S12. Transcription factors regulate the expression of *daf-16a* and *daf-16d/f*.

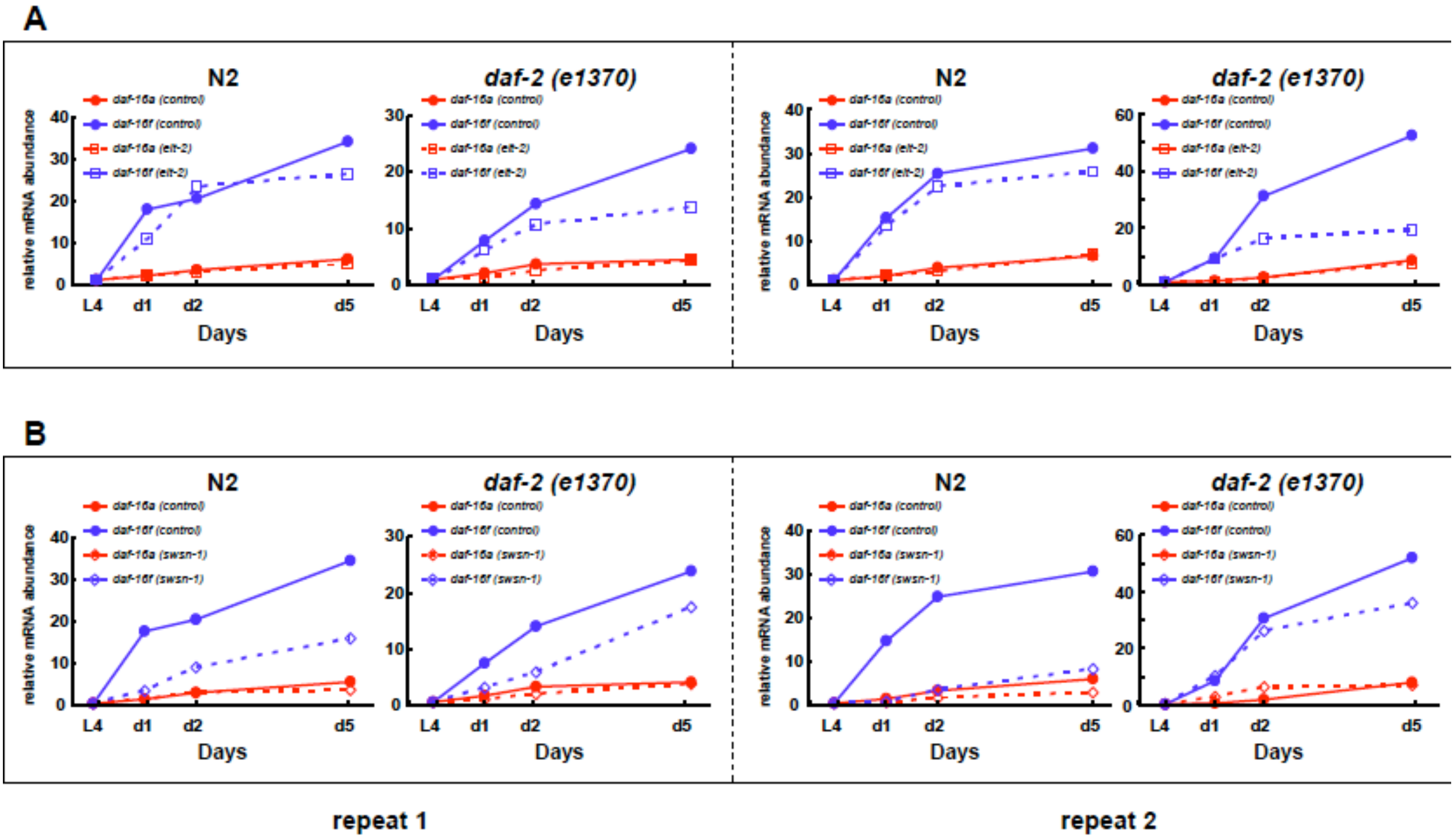

Figure S13. Effect by RNAi of GATA transcription factors on *Pdaf-16d/f::gfp* expression.

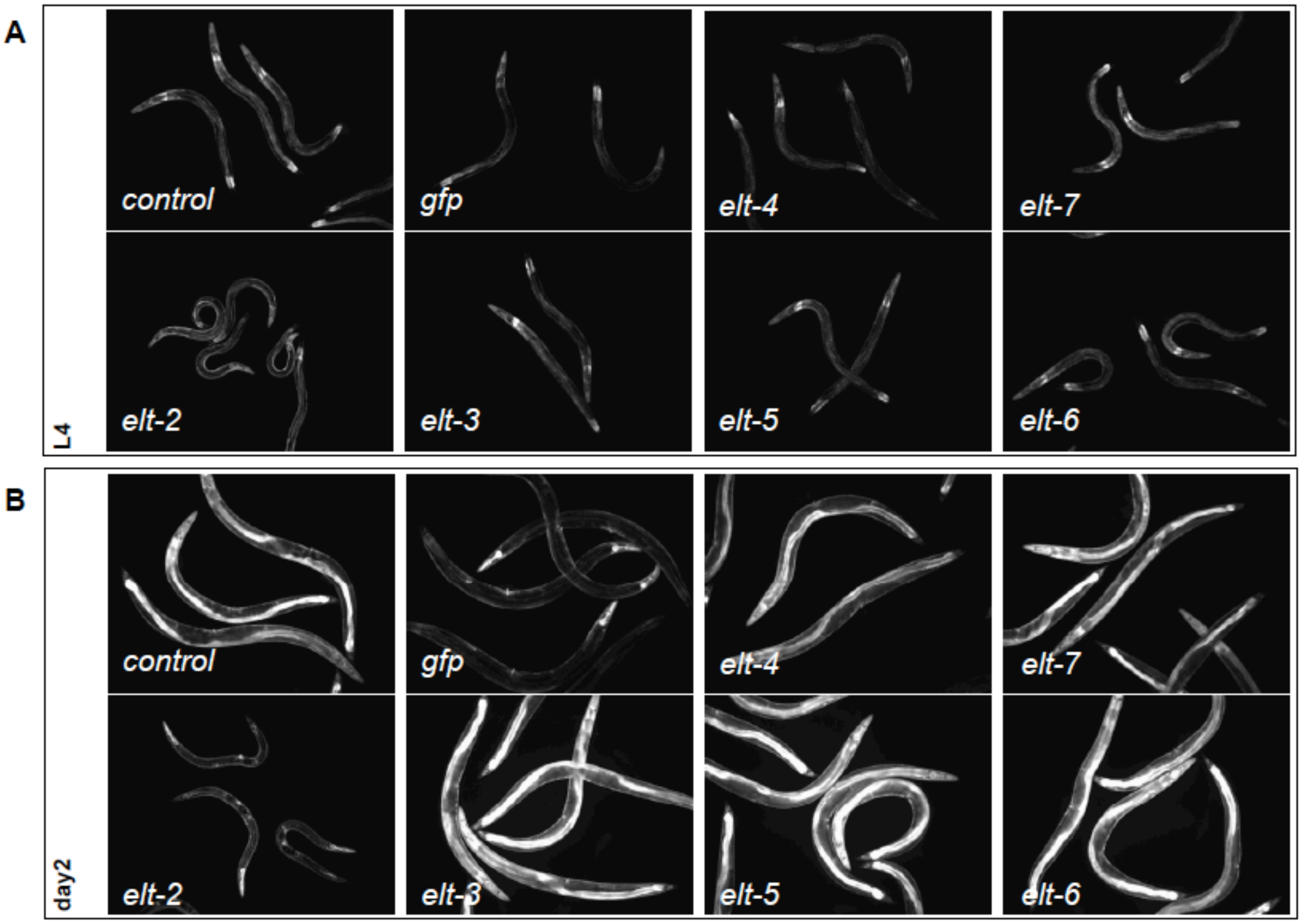

Figure S14. Effect by RNAi of SWI/SNF complex components on *Pdaf-16d/f::gfp* expression.

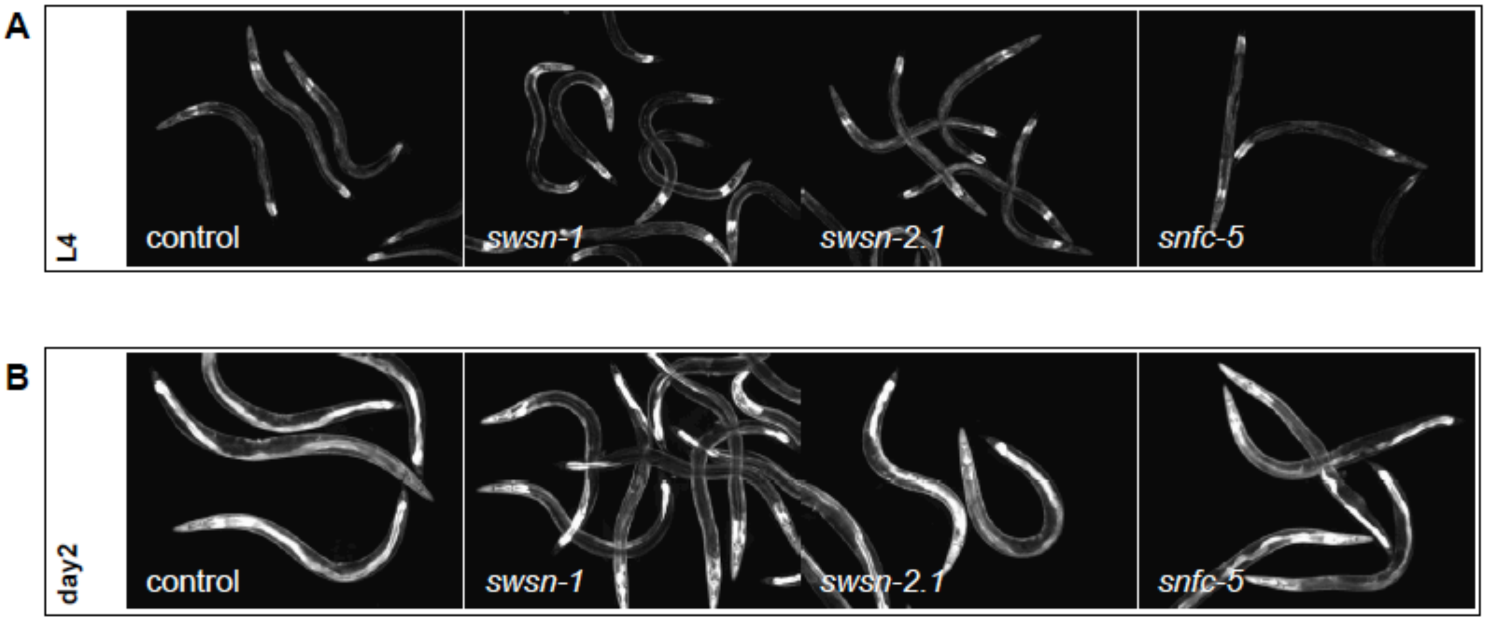

.

Figure S15: Lack of an effect of FUDR on lifespan.

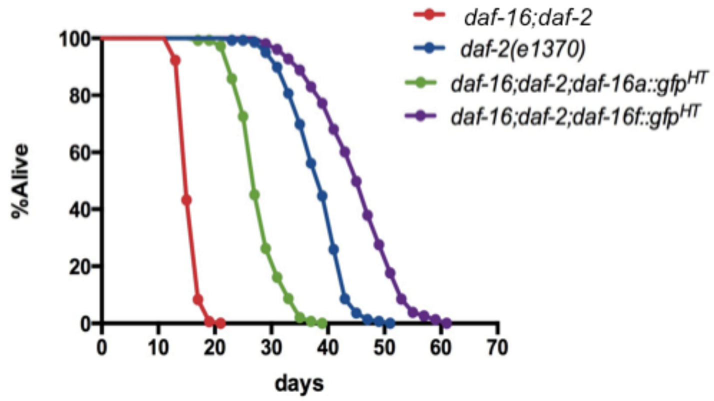

With FUDR

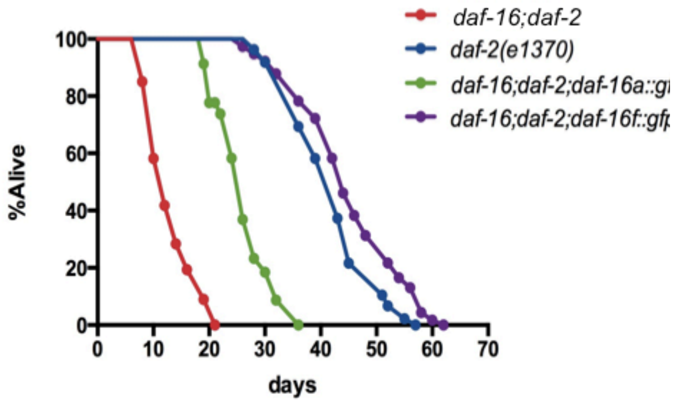

Without FUDR

Table S1 : Analysis of the ESTs that correspond to *daf-16* isoforms

			Available cDNA sequences				
	**# of cDNAs**	**Wormbase identity**	**Clone/accession**	**5' EST**	**3' EST**	**mRNA**	***note***
**Unique to *daf-16f***	1	R13H8.f	KZ41_1781086	5			
**Common to *daf-16f* and d:**	2	R13H8.f/d	yk1377b02	5	3		
			yk359c8	5	3		
**Unique to *daf-16a* (a1 and a2 isoforms are reported together):**	11	R13H8.b/c	OSTF020B8_1	5			a1 = R13H8.b
			OSTF020D8_1	5			a1 = R13H8.b
			yk1204c09	5	3		a1 = R13H8.b
			yk1008b03	5	3		a1 = R13H8.b
			AF020343			mRNA	a1 = R13H8.b
			IST_WI5_47437	5			a2 = R13H8.c
			yk31f10	5	3		a2 = R13H8.c
			AF020342			mRNA	a2 = R13H8.c
			yk1337d04	5	3		a1/a2 = R13H8.b/c
			yk1006c10	5	3		a1/a2 = R13H8.b/c
			yk13f11	5	3		a1/a2 = R13H8.b/c
**Common to *daf-16f*, d and a:**	5	R13H8.f/d/b/c	yk350a5	5			
			yk1598c08	5	3		
			KZ41_1785504	5	3		
			yk1564b12	5	3		
			yk1556f11	5	3		
**Unique to *daf-16b*:**	6	R13H8.a	yk572a12	5			
			yk632g3	5			
			OSTF020E10_1	5			
			yk294b10	5			
			yk274c4	5	3		
			AF020344			mRNA	
			OSTR020E10_1	3			
			OSTR020D8_1	3			
			OSTR020B8_1	3			
			yk14d11	3			
			yk178c8	5			
			yk1019c02	5	3		
			yk1196f06	5	3		
			yk1044b11	5	3		
			yk1048e05	5	3		
			yk32f8	5	3		
**Unnamed isoform**	1	R13H8.g	yk1160e04	5	3		

Table S2. Comparative level of *daf-16* isoform transcript

Comparative level of *daf-16* isoform transcript			
**Genotype**	***daf-16a***	***daf-16f***	**generated from** [ref]
daf-2(e1370)	**1 (control)**	**1 (control)**	
daf-16(mgDf50);daf-2(e1370)	**N. D.**	**N. D.**	
daf-16(mgDf50);daf-2(e1370);daf-16a::gfp^{HT}	**1.67 ± 0.17**	**N. D.**	**In this study**
daf-16(mgDf50);daf-2(e1370);daf-16a::gfp^{CF}	**10.19 ± 0.16**	**N. D.**	**Lin et al. [7]**
daf-16(mgDf50);daf-2(e1370);daf-16a::gfp^{TJ}	**22.95 ± 1.63**	**N. D.**	**Hederson et al. [8]**
daf-16(mgDf50);daf-2(e1370);daf-16a::gfp^{GR}	**432.85 ± 29.44**	**N. D.**	**Padmanabhan et al. [9]**
daf-16(mgDf50);daf-2(e1370);daf-16d/f::gfp^{HT}	**N. D.**	**4.57 ± 1.11**	**Kwon et al. [2]**

N. D., not detected

Table S3. Lifespan analysis of *daf-16* isoform transgenic strains

**Lifespan analysis of *daf-16* isoform transgenic strains**				
**Background**	***daf-16* isoform**	**Mean LS ± SEM (days)**	**Number of worms**	**P value vs control**
**exp.1**[a]				
**N2**	***daf-16*$^{+}$*(WT)***	**21.1 ± 0.4**	**59**	**control**
daf-16(mgDf50)	**Null**	**15.0 ± 0.1**	**144**	**< 0.0001**
	***daf-16a::gfp*^{HT}**	**19.4 ± 0.3**	**103**	**0.0007**
	***daf-16a::gfp*^{CF}**	**23.3 ± 0.3**	**126**	**< 0.0001**
	***daf-16a::gfp*^{TJ}**	**22.2 ± 0.3**	**118**	**0.0215**
	***daf-16a::gfp*^{GR}**	**27.5 ± 0.2**	**131**	**< 0.0001**
	***daf-16d/f::gfp*^{HT}**	**20.9 ± 0.4**	**58**	**0.7682**
daf-2(e1370)	***daf-16*$^{+}$*(WT)***	**38.5 ± 0.4**	**139**	**control**
daf-16(mgDf50);	**Null**	**15.9 ± 0.1**	**155**	**< 0.0001**
daf-2(e1370)	***daf-16a::gfp*^{HT}**	**28.1 ± 0.3**	**149**	**< 0.0001**
	***daf-16a::gfp*^{CF}**	**40.9 ± 0.4**	**155**	**< 0.0001**
	***daf-16a::gfp*^{TJ}**	**41.0 ± 0.4**	**157**	**< 0.0001**
	***daf-16a::gfp*^{GR}**	**27.8 ± 0.3**	**136**	**< 0.0001**
	***daf-16d/f::gfp*^{HT}**	**45.3 ± 0.6**	**153**	**< 0.0001**
**exp.2**				
**N2**	***daf-16*$^{+}$*(WT)***	**18.6 ± 0.3**	**114**	**control**
daf-16(mgDf50)	**Null**	**13.6 ± 0.2**	**137**	**< 0.0001**
	***daf-16a::gfp*^{HT}**	**17.3 ± 0.3**	**56**	**0.0006**
	***daf-16a::gfp*^{CF}**	**23.6 ± 0.3**	**127**	**< 0.0001**
	***daf-16a::gfp*^{TJ}**	**22.2 ± 0.3**	**114**	**< 0.0001**
	***daf-16a::gfp*^{GR}**	**27.8 ± 0.3**	**149**	**< 0.0001**
	***daf-16d/f::gfp*^{HT}**	**19.4 ± 0.5**	**59**	**0.029**
daf-2(e1370)	***daf-16*$^{+}$*(WT)***	**38.1 ± 0.5**	**111**	**control**
daf-16(mgDf50);	**Null**	**15.2 ± 0.1**	**141**	**< 0.0001**
daf-2(e1370)	***daf-16a::gfp*^{HT}**	**26.5 ± 0.3**	**141**	**< 0.0001**
	***daf-16a::gfp*^{CF}**	**43.2 ± 0.4**	**161**	**< 0.0001**
	***daf-16a::gfp*^{TJ}**	**39.2 ± 0.4**	**154**	**0.1965**
	***daf-16a::gfp*^{GR}**	**26.6 ± 0.3**	**130**	**< 0.0001**
	***daf-16d/f::gfp*^{HT}**	**44.9 ± 0.5**	**176**	**< 0.0001**
**exp.3**				
**N2**	***daf-16*$^{+}$*(WT)***	**19.9 ± 0.3**	**128**	**control**
daf-16(mgDf50)	**Null**	**15.4 ± 0.1**	**153**	**< 0.0001**
	***daf-16a::gfp*^{HT}**	**18.8 ± 0.2**	**238**	**< 0.0001**
	***daf-16a::gfp*^{CF}**	**19.8 ± 0.2**	**175**	**0.4561**
	***daf-16a::gfp*^{TJ}**	**21.3 ± 0.3**	**127**	**< 0.0001**
	***daf-16a::gfp*^{GR}**	**26.3 ± 0.2**	**151**	**< 0.0001**
	***daf-16d/f::gfp*^{HT}**	**20.2 ± 0.3**	**165**	**0.0809**
daf-2(e1370)	***daf-16*$^{+}$*(WT)***	**38.6 ± 0.5**	**163**	**control**
daf-16(mgDf50);	**Null**	**16.1 ± 0.1**	**187**	**< 0.0001**

daf-2(e1370)	***daf-16a::gfp*** ^{HT}	**31.9 ± 0.3**	**169**	**< 0.0001**
	daf-16a::gfp ^{CF}	**33.5 ± 0.3**	**177**	**< 0.0001**
	daf-16a::gfp ^{TJ}	**36.1 ± 0.3**	**180**	**< 0.0001**
	daf-16a::gfp ^{GR}	**28.9 ± 0.3**	**203**	**< 0.0001**
	daf-16d/f::gfp ^{HT}	**42.6 ± 0.5**	**191**	**< 0.0001**

LS= lifespan; [a] indicates experiment depicted in Figure 2B and 2C.

Table S3. Lifespan analysis of *daf-16* isoform transgenic strains (continued)

Table S4. Lifespan analysis of *daf-16* isoform transgenic strains

**Lifespan analysis of *daf-16* isoform transgenic strains**				
**Background**	***daf-16* isoform**	**Mean LS ± SEM (days)**	**Number of worms**	**P value vs control**
**exp.1**				
daf-2(e1368)	***daf-16$^+$(WT)***	**33.6 ± 0.8**	**67**	**control**
daf-16(mgDf50); daf-2(e1368)	**Null**	**15.0 ± 0.2**	**75**	**< 0.0001**
	daf-16a::gfp^{HT}	**25.3 ± 0.4**	**91**	**< 0.0001**
	daf-16d/f::gfp^{HT}	**27.7 ± 0.4**	**102**	**< 0.0001**
**exp.2**				
daf-2(e1368)	***daf-16$^+$(WT)***	**33.6 ± 0.7**	**104**	**control**
daf-16(mgDf50); daf-2(e1368)	**Null**	**13.3 ± 0.2**	**89**	**< 0.0001**
	daf-16a::gfp^{HT}	**22.2 ± 0.4**	**74**	**< 0.0001**
	daf-16d/f::gfp^{HT}	**27.7 ± 0.5**	**83**	**< 0.0001**
**exp.3[a]**				
daf-2(e1368)	***daf-16$^+$(WT)***	**29.8 ± 0.4**	**101**	**control**
daf-16(mgDf50); daf-2(e1368)	**Null**	**15.5 ± 0.2**	**100**	**< 0.0001**
	daf-16a::gfp^{HT}	**22.2 ± 0.2**	**195**	**< 0.0001**
	daf-16d/f::gfp^{HT}	**27.3 ± 0.3**	**215**	**< 0.0001**

LS, lifespan

[a] indicates experiment depicted in Figure 2D.

Table S5. Lifespan analysis of serially diluted *daf-16* RNAi

**Lifespan analysis of serially diluted *daf-16* RNAi**				
**Background**	**RNAi$^{(dilution)}$**	**Mean LS ± SEM (days)**	**Number of worms**	**P value vs control**
**exp.1[a]**				
daf-16(mgDf50); daf-2(e1370)	**control**	**14.0 ± 0.2**	**151**	**control**
	daf-16	**14.4 ± 0.2**	**172**	**0.0875**
daf-16(mgDf50); daf-2(e1370); daf-16d/f::gfp	**control**	**41.8 ± 0.4**	**288**	**control**
	daf-16	**17.6 ± 0.2**	**226**	**< 0.0001**
	***daf-16*$^{(1/1)*}$**	**17.3 ± 0.2**	**171**	**< 0.0001**
	***daf-16*$^{(1/4)*}$**	**21.9 ± 0.4**	**242**	**< 0.0001**
	***daf-16*$^{(1/8)*}$**	**26.4 ± 0.4**	**257**	**< 0.0001**
	***daf-16*$^{(1/16)*}$**	**30.4 ± 0.3**	**226**	**< 0.0001**
	***daf-16*$^{(1/32)*}$**	**34.2 ± 0.4**	**264**	**< 0.0001**
	***daf-16*$^{(1/64)*}$**	**38.5 ± 0.4**	**255**	**< 0.0001**
**exp.2**				
daf-16(mgDf50); daf-2(e1370)	**control**	**14.1 ± 0.1**	**131**	**control**
	daf-16	**14.3 ± 0.1**	**148**	**0.2444**
daf-16(mgDf50); daf-2(e1370); daf-16f::gfp	**control**	**54.1 ± 0.5**	**156**	**control**
	daf-16	**17.0 ± 0.3**	**77**	**< 0.0001**
	***daf-16*$^{(1/1)*}$**	**16.8 ± 0.2**	**109**	**< 0.0001**
	***daf-16*$^{(1/2)*}$**	**17.3 ± 0.3**	**104**	**< 0.0001**
	***daf-16*$^{(1/4)*}$**	**18.4 ± 0.3**	**102**	**< 0.0001**
	***daf-16*$^{(1/8)*}$**	**19.6 ± 0.4**	**117**	**< 0.0001**
	***daf-16*$^{(1/16)*}$**	**27.0 ± 0.8**	**91**	**< 0.0001**

( ) indicates dilution rate; $^{(\textit{daf-16}\ \text{RNAi}/\textit{daf-16}\ \text{RNAi + control RNAi})}$

*** indicates temporal treatment of *daf-16* RNAi.**

[a] indicates experiment depicted in Figure 3F.

LS, lifespan

Table S7. Primer sequences used in this study

**Primer sequences used in this study**			
**Primer name**	***Gene amplified***	**Used for**	**SEQUENCE (5’-3’)**
**8.1cspf5qRT**	***daf-16a***	**Q-RT PCR**	**CACCGGATGATGTGATGATG**
**8.1cspf3qRT**	***daf-16a***	**Q-RT PCR**	**CTCCCGTATAGGTCAGCATC**
8.1aspf5qRT	***daf-16b***	**Q-RT PCR**	**CCTATTCGGATATCATTGCC**
**8.1aspf3qRT**	***daf-16b***	**Q-RT PCR**	**GGATCGAGTTCTTCCATCCG**
**8.1dspf5qRT**	***daf-16d/f***	**Q-RT PCR**	**CAATCTCGACCTCCATCAAC**
**8.1dspf3qRT**	***daf-16d/f***	**Q-RT PCR**	**CCCGTATAGGCTAGTTCTTC**
**8.1ORFcom5**	***total daf-16***	**Q-RT PCR**	**AAGCCGATTAAGACGGAACC**
**8.1ORFcom3**	***total daf-16***	**Q-RT PCR**	**GTAGTGGCATTGGCTTGAAG**
**acts-5**	***Actin***	**Q-RT PCR**	**CTCTTGCCCCATCAACCATG**
**acts-3**	***Actin***	**Q-RT PCR**	**CTTGCTTGGAGATCCACATC**
**elt-2 RT fw2**	***elt-2***	**Q-RT PCR**	**GGGTTGATGATGGTTCCAAAC**
**elt-2 RT rv2**	***elt-2***	**Q-RT PCR**	**TTTCATCATCCTGAACTGGC**
**psa-1 RT fw1**	***swsn-1***	**Q-RT PCR**	**GAAGTGCCGAAAGGAAAGGAA**
**psa-1 RT rv1**	***swsn-1***	**Q-RT PCR**	**TTTCCTTCGGCGAGTTGTGG**
**Pdaf-16df5**	***daf-16d/f***	**Translational gfp fusion**	**TATTGCATGCCGTAGCTTAAAG**
**Pdaf16df3 BamHI**	***daf-16d/f***	**Translational gfp fusion**	**TCCGGGATCCGGCCTTGCCGGTT**[illegible]
**Pdaf16bc5 SalI**	***daf-16a***	**Translational gfp fusion**	**CATTGTCGACGCCGCGCCCCATG**
**Pdaf16bc3 BamHI**	***daf-16a***	**Translational gfp fusion**	**TTCGGGATCCTCAGCCAAAGACG**<br>**AC**
**16D RNAi5**	***daf-16d/f***	**RNAi cloning**	**AACTGAAGCTTGATTCGCCGCTA**[illegible]<br>**C**
**16D RNAi3**	***daf-16d/f***	**RNAi cloning**	**CCCGTGCTAGCTAGTTCTTCCGC**
**DAF16 INT UP**	***daf-16***	**detecting deletion**	**CAATGAGCAATGTGGACAGC**
**DAF16 INT down**	***daf-16***	**detecting deletion**	**CCGTCTGGTCGTTGTCTTTT**

Table S8. List of Strains used in this study

Strain Name	Genotype	Reference
	**Wild type bristol**	
**CB1370**	***daf-2(e1370)***	**Kenyon et al. 1993**
**GR1307**	***daf-16(mgDf50)***	**Ogg et al. 1997**
**HT1890**	***daf-16(mgDf50); daf-2(e1370)***	
**HT2001**	***daf-16(mgDf50);unc-119(ed3); daf16a1::GFP unc-119[LpIs24]***	**Kwon et al. 2010**
~~CF1407~~	***daf-16(mgDf50);muIs71 [pKL99(daf-16Ap::GFP::daf-16A(bKO)) + pRF4(rol-6)]***	**Lin et al. 2001**
**TJ356**	***daf-16(mgDf50);* zIs356**	**Henderson et al. 2001**
**RX 86**	***daf-16a::gfp***	**Padmanabhan et a[illegible] 2008**
**HT2058**	***daf-16(mgDf50); daf-2(e1370); unc-119(ed3); daf16a1::a::GFP unc-119[LpIs24]***	**This manuscript**
**HT2025**	***daf-16(mgDf50);daf-2(e1370);muIs71 [pKL99(daf-16Ap::GFP::daf-16A(bKO)) +pRF4(rol-6)]***	**This manuscript**
**HT2022**	***daf-16(mgDf50);daf-2(e1370);* zIs356**	**This manuscript**
**HT 2055**	***daf-16; daf-2; daf-16a::gfp*** ^{GR}	**This manuscript**
**DR1572**	***daf-2(e1368)***	
**HT2006**	***daf-16(mgDf50);daf-2(e1368)***	**This Manuscript**
**HT1970**	***daf-16(mgDf50);unc-119(ed3); daf-16d/f::GFP unc-119[LpIs14]***	**Kwon et al. 2010**
**HT1883**	***daf-16(mgDf50);daf-2(e1370);unc-119(ed3); daf-16d/f::GFP unc-119[LpIs14]***	**Kwon et al. 2010**
**HT2014**	***unc-119(ed3);Pdaf-16a::gfp unc-119[LpIs27]***	**This manuscript**
**HT1992**	***unc-119(ed3);Pdaf-16d/f::gfp unc-119[LpIs23]***	**This manuscript**
**HT2063**	***daf-16(mgDf50); daf-2(e1368); unc-119(ed3); daf16a1::a::GFP unc-119[LpIs24]***	**This manuscript**
**HT2040**	***daf-16(mgDf50); daf-2(e1368); unc-119(ed3); daf-16d/f::GFP unc-119[LpIs14]***	**This manuscript**
